# Posttranslational ISGylation of NLRP3 by HERC enzymes facilitates inflammasome activation in models of inflammation

**DOI:** 10.1172/JCI161935

**Published:** 2023-10-16

**Authors:** Ying Qin, Xintong Meng, Mengge Wang, Wenbo Liang, Rong Xu, Jingchunyu Chen, Hui Song, Yue Fu, Jingxin Li, Chengjiang Gao, Mutian Jia, Chunyuan Zhao, Wei Zhao

**Affiliations:** Key Laboratory for Experimental Teratology of the Chinese Ministry of Education and Key Laboratory of Infection and Immunity of Shandong Province, School of Basic Medical Science, Qilu Hospital, Cheeloo College of Medicine, Shandong University, Jinan, China.

**Keywords:** Immunology, Inflammation, Cellular immune response, Innate immunity

## Abstract

The NOD-, LRR-, and pyrin domain–containing protein 3 (NLRP3) inflammasome is a crucial component of the innate immune system that initiates inflammatory responses. Posttranslational modifications (PTMs) of NLRP3, including ubiquitination and phosphorylation, control inflammasome activation and determine the intensity of inflammation. However, the role of other PTMs in controlling NLRP3 inflammasome activation remains unclear. This study found that TLR priming induced NLRP3 ISGylation (a type of PTM in which ISG15 covalently binds to the target protein) to stabilize the NLRP3 protein. Viral infection, represented by SARS-COV-2 infection, and type I IFNs induced expression of ISG15 and the predominant E3 ISGylation ligases HECT domain- and RCC1-like domain–containing proteins (HERCs; HERC5 in humans and HERC6 in mice). HERCs promoted NLRP3 ISGylation and inhibited K48-linked ubiquitination and proteasomal degradation, resulting in the enhancement of NLRP3 inflammasome activation. Concordantly, *Herc6* deficiency ameliorated NLRP3-dependent inflammation as well as hyperinflammation caused by viral infection. The results illustrate the mechanism by which type I IFNs responses control inflammasome activation and viral infection–induced aberrant NLRP3 activation. This work identifies ISGylation as a PTM of NLRP3, revealing a priming target that modulates NLRP3-dependent immunopathology.

## Graphical abstract

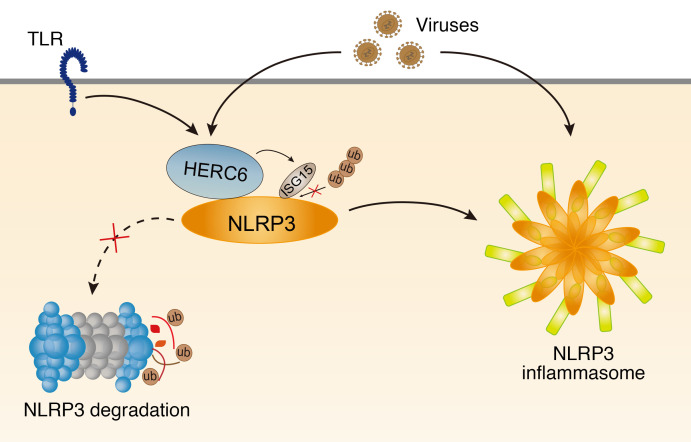

## Introduction

The NOD-, LRR-, and pyrin domain–containing protein 3 (NLRP3) inflammasome is a vital component of the innate immune system that senses both pathogen-associated molecular patterns (PAMPs) from microbes and host-derived damage-associated molecular patterns (DAMPs), and then initiates inflammatory responses (1–4). Upon activation, NLRP3 recruits the adaptor apoptosis-associated speck-like protein containing a CARD (ASC) and the cysteine protease pro–caspase-1 (pro-CASP1) to form the inflammasome. NLRP3 inflammasome activation induces pro-CASP1 cleavage to its active forms (p10 and p20), which further cleave pro–IL-1β, pro–IL-18, and gasdermin D (GSDMD). The cleaved GSDMD then forms pores in the plasma membrane, through which the biologically active forms of cytokines (IL-1β and IL-18) are released and pyroptosis is induced (5). Optimal activation of the NLRP3 inflammasome is beneficial for the host’s ability to eliminate invading pathogens efficiently and quickly (1, 6). However, aberrant NLRP3 inflammasome activation has been implicated in various inflammation-associated diseases, such as diabetes, atherosclerosis, obesity, cancer, and Alzheimer’s disease (7).

INF-induced 15 kDa protein (ISG15), a ubiquitin-like protein, has been implicated as a central player in host defense against pathogens (8). ISG15 can function as a cytokine, interact with various intracellular proteins, or covalently bind other proteins via ISGylation to exert its immunomodulatory activities (8). Extracellular ISG15 induces NK cell proliferation and IFN-γ production (9, 10). Intracellular ISG15 prevents type I IFN overamplification in an ISGylation-independent manner (11). ISGylation is a 3-step enzymatic cascade that requires the E1 enzyme ubiquitin-like modifier–activating enzyme 7 (UBA7; also known as UBE1L); the E2 enzyme ubiquitin/ISG15-conjugating enzyme E2 L6 (UBE2L6; also known as UBCH8); and the E3 ligase, which in humans is mainly HECT domain– and RCC1-like domain–containing protein 5 (HERC5), whereas in mice HERC6 performs this function (8). ISGylation plays a crucial role in host defense by regulating IFN response. For example, ISGylation of melanoma differentiation–associated protein 5 (MDA5) is required for its activation and restricts virus replication. Furthermore, viral infection induces expression of ISGylation enzymes, including UBE1L, UBCH8, ISG15, and HERC5, suggesting that ISGylation is involved in host defenses against viruses (12).

Recently, ISGylation rather than free ISG15 was found to exacerbate the secretion of multiple proinflammatory cytokines (including IL-6 and IL-1β) and chemokines (including CCL2), potentially contributing to the cytokine storm of COVID-19 (13). Higher levels of IL-18 and CASP p20 are associated with disease severity and poor clinical outcomes of COVID-19, indicating that inflammasomes participate in the pathophysiology of viral diseases (14). Therefore, targeting of NLRP3 inflammasome may constitute a potential treatment strategy for immunopathology mediated by chronic SARS-CoV-2 infection in vivo (15). However, the potential role of ISGylation in NLRP3 inflammasome activation and viral infections caused excessive inflammation remains unknown.

In the present study, we identified HERCs (HERC5 in humans and HERC6 in mice) as enhancers of inflammasome activation that selectively target NLRP3. HERCs facilitate ISGylation of NLRP3, block its K48-linked polyubiquitination, and inhibit its proteasomal degradation. Concordantly, *Herc6* deficiency ameliorates NLRP3-dependent inflammation and hyperinflammation caused by viral infection. The results presented here elucidate the crosstalk between type I IFNs and inflammatory responses and uncover a mechanism by which viral infection causes NLRP3 hyperactivation. ISGylation is identified as a posttranslational modification (PTM) of NLRP3, revealing a priming target that modulates NLRP3-dependent immunopathology.

## Results

### HERCs interact with NLRP3.

**Figure 1:**
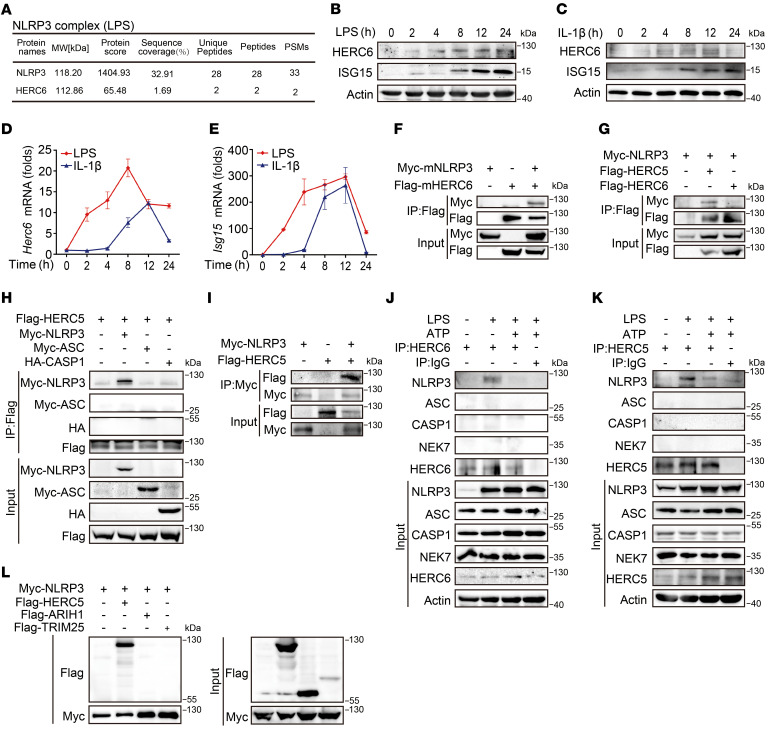
HERCs interact with NLRP3. (**A**) Identification of HERC6 as a potential NLRP3 interactor in LPS-stimulated mouse PMs by mass spectrometry. (**B** and **C**) Immunoblot analysis of HERC6 and ISG15 expression in LPS-stimulated (**B**) or mIL-1β–stimulated (**C**) mouse PMs. (**D** and **E**) RT-PCR analysis of *Herc6* (**D**) or *Isg15* (**E**) mRNA in LPS-stimulated or mIL-1β–stimulated mouse PMs. All data are presented as the mean ± SD. (**F**) Co-IP analysis of the association between mHERC6 and mNLRP3 in HEK293T cells transfected with the indicated plasmids. (**G**) Co-IP analysis of the association between hNLRP3 and hHERC5 or hHERC6 in HEK293T cells transfected with the indicated plasmids. (**H**) Co-IP analysis of the association between HERC5 and NLRP3, ASC, or CASP1 in HEK293T cells transfected with the indicated plasmids. (**I**) Myc-NLRP3 and Flag-HERC5 were obtained by in vitro transcription and translation. The interaction between NLRP3 and HERC5 was assayed by mixing recombinant Myc-NLRP3 and Flag-HERC5, followed by co-IP with Myc antibody and immunoblot analysis with Flag or Myc antibody. (**J**) Co-IP analysis of the endogenous association between HERC6 and NLRP3, ASC, CASP1, or NEK7 in LPS-stimulated or LPS-primed and ATP-activated mouse PMs. (**K**) Co-IP analysis of the endogenous association between HERC5 and NLRP3, ASC, CASP1, or NEK7 in LPS-stimulated or LPS-primed and ATP-activated THP-1 cells. (**L**) Co-IP analysis of the association between NLRP3 and HERC5, ARIH1, or TRIM25 in HEK293T cells transfected with the indicated plasmids. Similar results were obtained from 3 independent experiments.

To identify potential molecules involved in NLRP3 activation, we incubated a specific anti-NLRP3 antibody with total cell lysates from mouse primary peritoneal macrophages (PMs) stimulated with LPS. The coimmunoprecipitated complex was separated by SDS-PAGE. The E3 ISGylation ligase HERC6 was identified as a potential NLRP3 interactor by liquid chromatography coupled with tandem mass spectrometry (Figure 1A). In addition, both LPS and IL-1β induced HERC6 and ISG15 expression (Figure 1, B–E).

The interactions between HERC6 and NLRP3 were then examined. HERC6 and NLRP3 were cotransfected into HEK293T cells, and mHERC6 was immunoprecipitated with mNLRP3 (Figure 1F). In human cells, HERC5 (hHERC5) is the main ISG15 E3 ligase (16), whereas human HERC6 (hHERC6) lacks any ISG15-conjugating activity. We observed that LPS stimulation induced hHERC5 expression in THP-1 cells (Supplemental Figure 1, A and B; supplemental material available online with this article; https://doi.org/10.1172/JCI161935DS1). hHERC5, but not hHERC6, interacted with hNLRP3 in HEK293T cells (Figure 1G). Moreover, ASC and CASP1 did not interact with HERC6 (Figure 1H). In vitro binding assays demonstrated that HERC5 directly interacted with NLRP3 (Figure 1I). The interactions between HERCs and NLRP3 under physiological conditions were then investigated. An association between HERC6 and NLRP3 was detected in LPS-stimulated mouse PMs (Figure 1J and Supplemental Figure 1C). Similarly, in human THP-1 cells, HERC5 interacted with NLRP3 after LPS stimulation (Figure 1K). However, ASC, CASP1, NEK7, and NLRC4 did not interact with HERCs in LPS-stimulated macrophages, with or without ATP stimulation (Figure 1, J and K, and Supplemental Figure 1D). Of note, ATP treatment diminished the interaction between HERCs and NLRP3 (Figure 1, J and K, and Supplemental Figure 1C). Other E3 ISGylation ligases, including Ariadne-1 homolog (ARIH1) and TRIM25, did not interact with NLRP3 (Figure 1L). Collectively, these data indicated a dynamic interaction between HERCs (HERC5 in humans and HERC6 in mice) and NLRP3 during NLRP3 inflammasome activation.

### HERC6 selectively facilitates NLRP3 inflammasome activation.

**Figure 2:**
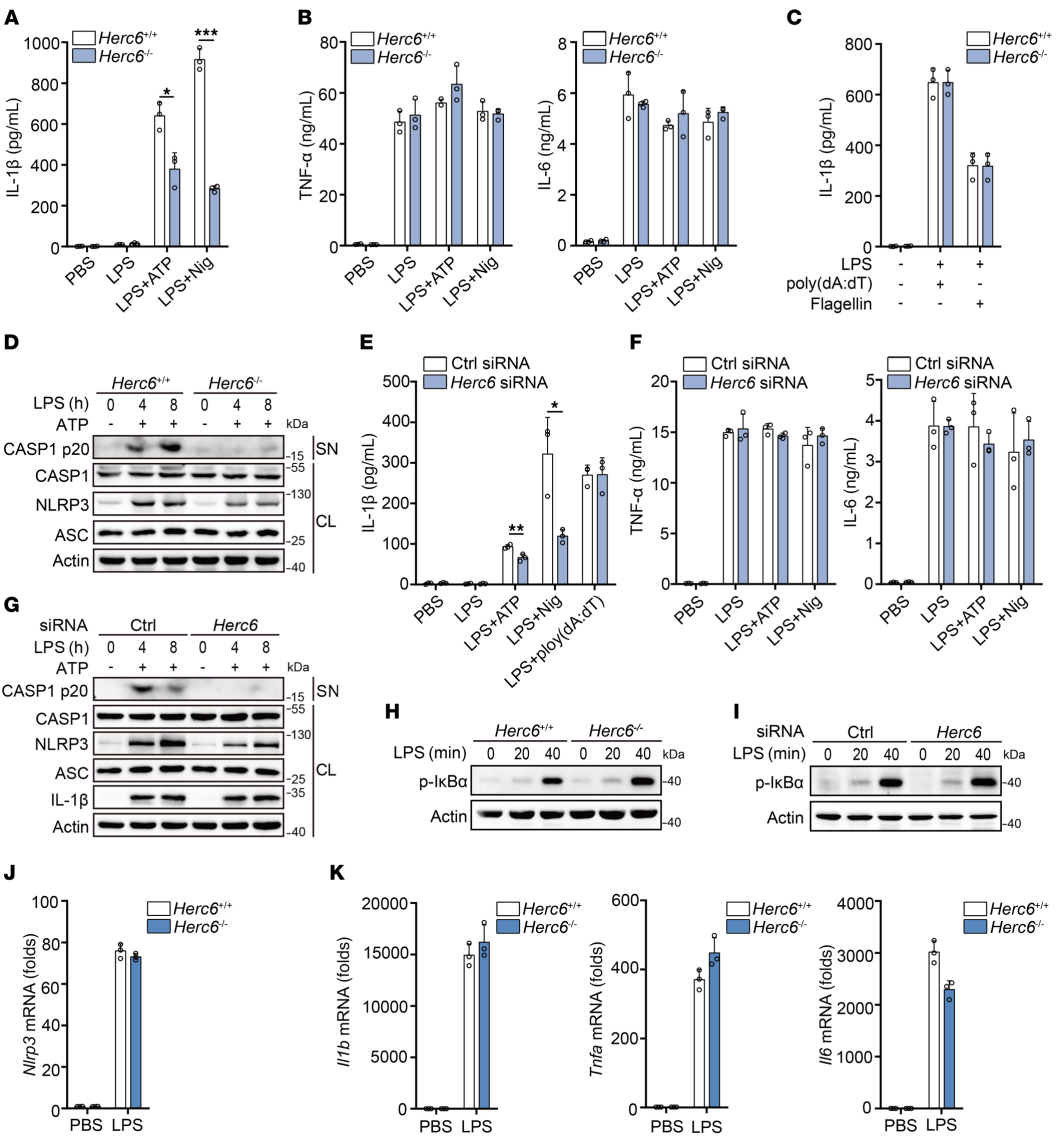
HERC6 selectively promotes NLRP3 inflammasome activation. (**A** and **B**) ELISA analysis of IL-1β, TNF-α, and IL-6 in supernatants of PMs from *Herc6*^+/+^ or *Herc6*^–/–^ mice following priming with LPS for 7 hours and stimulation with ATP or Nig for 1 hour (**A**: 2-tailed *t* test, *Herc6*^+/+^ vs. *Herc6*^–/–^, *P = 0.012074, ***P = 0.000040). (**C**) ELISA analysis of IL-1β in supernatants of PMs from *Herc6*^+/+^ or *Herc6*^–/–^ mice following LPS priming for 7 hours and stimulation with poly(dA:dT) or flagellin for 1 hour. (**D**) Immunoblot analysis of supernatants (SN) and cell lysates (CL) of PMs from *Herc6^+/+^* or *Herc6^–/–^* mice, following LPS priming and subsequent ATP stimulation for 1 hour. (**E**) ELISA analysis of IL-1β in supernatants from PMs transfected with control (Ctrl) siRNA or *Herc6* siRNA for 48 hours, followed by priming with LPS for 7 hours and stimulation with ATP, Nig, or poly(dA:dT) for 1 hour (2-tailed *t* test, Ctrl siRNA vs. *Herc6* siRNA, **P = 0.009794, *P = 0.018808). (**F**) ELISA analysis of TNF-α and IL-6 in supernatants from PMs transfected with Ctrl or *Herc6* siRNA for 48 hours, followed by priming with LPS for 7 hours and stimulation with ATP or Nig for 1 hour. (**G**) Immunoblot analysis of SN and CL of mouse PMs transfected with Ctrl siRNA or *Herc6* siRNA for 48 hours, followed by LPS priming and ATP stimulation for 1 hour. (**H** and **I**) Immunoblot analysis of p-IκBα in LPS-stimulated PMs from *Herc6*^+/+^ or *Herc6*^–/–^ mice (**H**), or PMs transfected with Ctrl or *Herc6* siRNA (**I**). (**J** and **K**) RT-PCR analysis of *Nlrp3* or *Il1b*, *Tnfa,* and *Il6* mRNA in LPS-stimulated PMs from *Herc6*^+/+^ or *Herc6*^–/–^ mice. All data are presented as the mean ± SD in **A**–**C**, **E**, **F**, **J**, and **K**. Similar results were obtained from 3 independent experiments.

Mouse PMs from both WT and *Herc6*-deficient mice were primed with LPS and then stimulated by the NLRP3 inflammasome activators ATP, nigericin (Nig), alum, and monosodium urate (MSU). *Herc6* deficiency markedly reduced NLRP3 inflammasome–induced IL-1β secretion but did not affect either TNF-α or IL-6 expression (Figure 2, A and B, and Supplemental Figure 2, A–D). However, IL-1β secretion stimulated by poly(dA:dT) (an absent in melanoma 2 [AIM2] inflammasome activator) and flagellin (an NLR family CARD domain–containing protein 4 [NLRC4] inflammasome activator) was not affected by *Herc6* deficiency (Figure 2C). *Herc6* deficiency also suppressed CASP1 cleavage following NLRP3 inflammasome activation but had no effect on CASP1 cleavage following NLRC4 or AIM2 inflammasome activation (Figure 2D and Supplemental Figure 2E). Meanwhile, we observed that *Herc6* deficiency markedly attenuated LPS-induced NLRP3 expression, with no effect on the expression of ASC or CASP1 (Figure 2D and Supplemental Figure 2E). Consistent with these findings, *Herc6* knockdown also reduced IL-1β secretion following ATP, Nig, alum, and MSU stimulation in LPS-primed mouse PMs, with no effect on TNF-α or IL-6 expression (Figure 2, E and F, and Supplemental Figure 2, F and G), and attenuated CASP1 cleavage following NLRP3 inflammasome activation (Figure 2G). However, *Herc6* knockdown did not affect poly(dA:dT)-induced IL-1β secretion (Figure 2E), or flagellin- or poly(dA:dT)-induced CASP1 cleavage (Supplemental Figure 2H). Overall, these data indicated that HERC6 selectively enhanced NLRP3 inflammasome activation.

Canonical NLRP3 inflammasome activation requires a priming step, which induces the expression of pro–IL-1β and NLRP3 by activating NF-κB. *Herc6* deficiency and knockdown did not affect NF-κB activation (Figure 2, H and I) or the mRNA expression of *Nlrp3*, *Il1b*, *Tnfa*, or *Il6* (Figure 2, J and K, and Supplemental Figure 2, I and J). These data indicated that HERC6 had no effect NF-κB activation or NLRP3 transcription.

### HERCs inhibit NLRP3 protein degradation.

**Figure 3:**
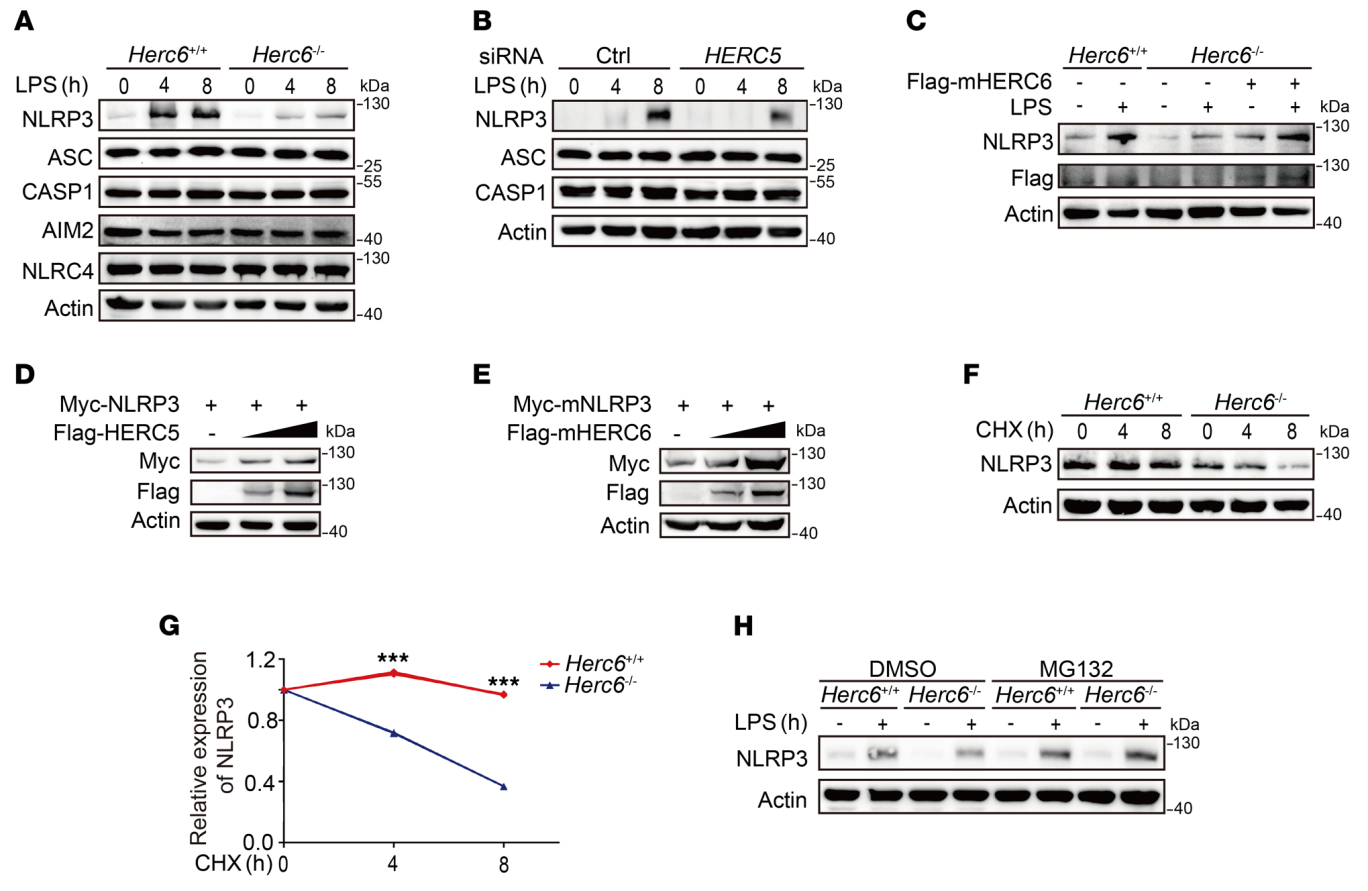
HERCs promote NLRP3 expression. (**A**) Immunoblot analysis of lysates of PMs from *Herc6*^+/+^ or *Herc6*^–/–^ mice, following LPS stimulation. (**B**) Immunoblot analysis of lysates of THP-1 cells transfected with Ctrl or *HERC5* siRNA for 48 hours, following LPS stimulation. (**C**) Immunoblot analysis of lysates from *Herc6*^+/+^ or *Herc6*^–/–^ MEFs transfected with Flag-mHERC6 or empty vector plasmid, following LPS stimulation for 8 hours. (**D**) Immunoblot analysis of lysates from HEK293T cells transfected with Myc-hNLRP3 and increasing amounts of Flag-hHERC5 plasmid. (**E**) Immunoblot analysis of lysates from HEK293T cells transfected with Myc-mNLRP3 and an increasing amounts of Flag-mHERC6 plasmid. (**F** and **G**) Immunoblot analysis of NLRP3 expression in PMs from *Herc6*^+/+^ or *Herc6*^–/–^ mice stimulated with LPS for 4 hours and then treated with cycloheximide (CHX) for the indicated times. NLRP3 expression was quantitated by measuring band intensities using ImageJ software. The values were normalized to actin. Data are represented as mean ± SD (2-tailed *t* test, *Herc6*^+/+^ vs. *Herc6*^–/–^, ***P = 1.43 × 10^–7^ and 1.80 × 10^–9^, at days 4 and 8 respectively). (**H**) Immunoblot analysis of NLRP3 expression in PMs from *Herc6*^+/+^ or *Herc6*^–/–^ mice stimulated with LPS for 4 hours, together with DMSO or MG132 treatment for an additional 4 hours. Similar results were obtained from 3 independent experiments.

The cellular NLRP3 protein level is vital for inflammasome assembly, and low quantities are considered rate-limiting for canonical NLRP3 inflammasome activation. *Herc6* deficiency markedly inhibited NLRP3 protein expression in mouse PMs, with no effect on the expression of ASC, CASP1, AIM2, or NLRC4 (Figure 3A). Similarly, *HERC5* knockdown in human THP-1 cells inhibited NLRP3 protein expression (Figure 3B and Supplemental Figure 3A) but did not affect *NLRP3* mRNA expression (Supplemental Figure 3B). Furthermore, overexpression of HERC6 or HERC5 restored NLRP3 protein levels in *Herc6*-deficient mouse embryonic fibroblasts (MEFs) (Figure 3C and Supplemental Figure 3C), and enhanced NLRP3 protein levels in a dose-dependent manner in HEK293T cells (Figure 3, D and E). To further investigate whether HERC6 could affect the protein degradation of NLRP3, we performed a cycloheximide (CHX) chase experiment. *Herc6* deficiency promoted significant NLRP3 degradation (Figure 3, F and G). MG132, a selective proteasome inhibitor, restored NLRP3 protein expression in LPS-stimulated *Herc6*-deficient macrophages (Figure 3H). These data collectively indicate that HERCs inhibit NLRP3 protein degradation by the proteasome.

### HERCs promote ISGylation of NLRP3.

**Figure 4:**
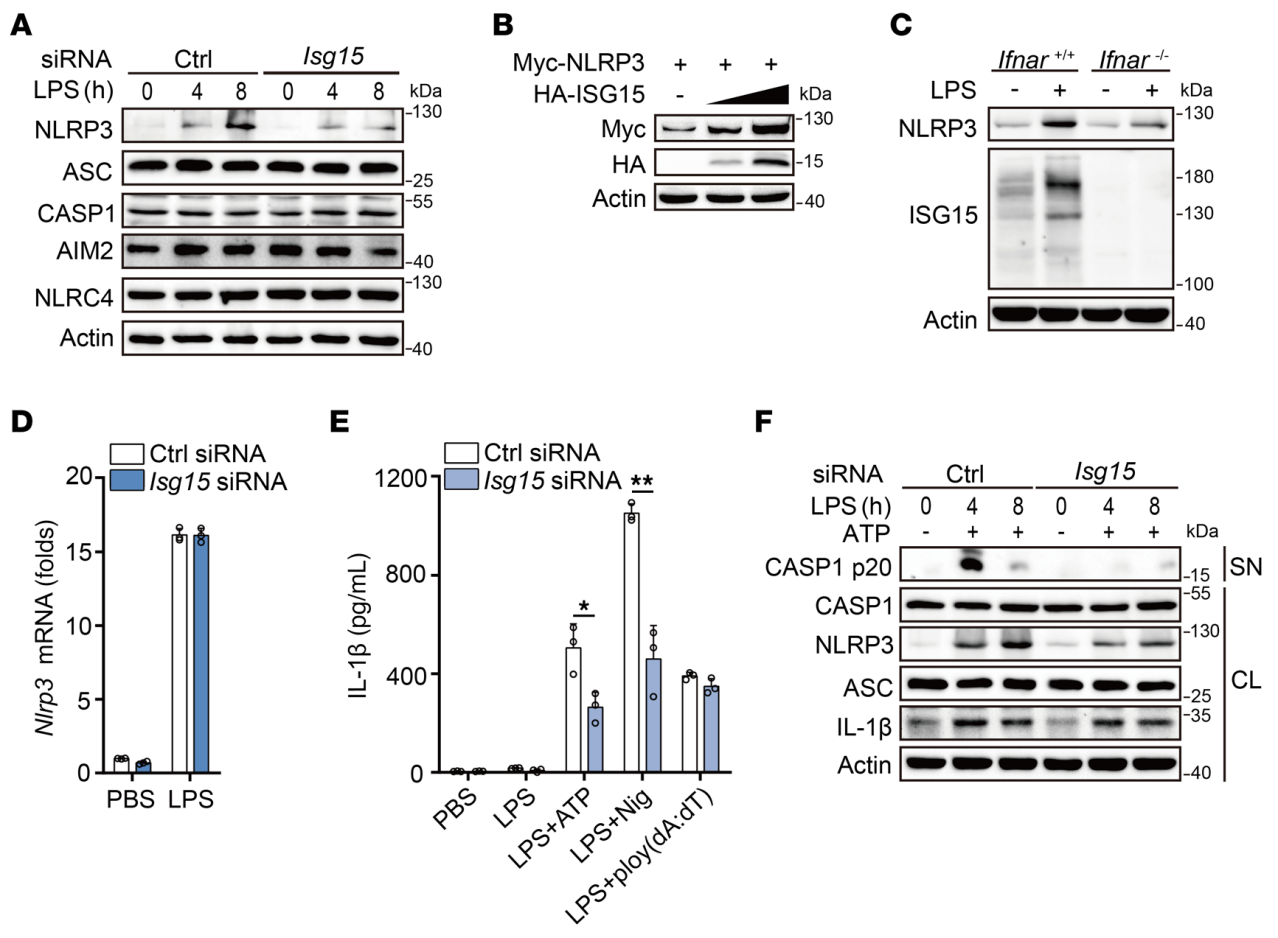
ISG15 enhances NLRP3 expression and inflammasome activation. (**A**) Immunoblot analysis of lysates of mouse PMs transfected with Ctrl or *Isg15* siRNA for 48 hours, following LPS stimulation. (**B**) Immunoblot analysis of lysates from HEK293T cells transfected with Myc-NLRP3 and increasing amounts of HA-ISG15 plasmid. (**C**) Immunoblot analysis of lysates of PMs from *Ifnar*^+/+^ or *Ifnar*^–/–^ mice, following LPS stimulation for 8 hours. (**D**) RT-PCR analysis of *Nlrp3* mRNA in LPS-stimulated mouse PMs transfected with Ctrl or *Isg15* siRNA for 48 hours. (**E**) ELISA analysis of IL-1β in supernatants from mouse PMs transfected with Ctrl or *Isg15* siRNA for 48 hours, followed by priming with LPS for 7 hours and subsequent stimulation with ATP, Nig, or poly(dA:dT) for 1 hour (2-tailed *t* test, Ctrl siRNA vs. *Isg15* siRNA, *P = 0.022242, **P = 0.001884). (**F**) Immunoblot analysis of SN and CL from mouse PMs transfected with Ctrl or *Isg15* siRNA for 48 hours, followed by LPS priming and subsequent ATP stimulation for 1 hour. All data are presented as mean ± SD in **D** and **E**. Similar results were obtained from 3 independent experiments.

To clarify the mechanism by which HERCs, as E3-ISGylation ligases, enhance NLRP3 inflammasome activation, we examined the potential effects of ISG15 on NLRP3 expression. *Isg15* knockdown inhibited NLRP3 protein expression in the PMs, with no influence on the expression of ASC, CASP1, AIM2, or NLRC4 (Figure 4A and Supplemental Figure 4A). Consistent with this finding, ISG15 overexpression enhanced NLRP3 expression in HEK293T cells (Figure 4B). In *Ifnar*-deficient PMs, LPS-induced NLRP3 protein expression was markedly decreased (Figure 4C). However, *Isg15* knockdown did not affect *Nlrp3* mRNA expression (Figure 4D).

Next, we examined the effect of ISG15 on inflammasome activation. *Isg15* knockdown attenuated ATP, Nig, alum, and MSU (Figure 4E and Supplemental Figure 4B), but not poly(dA:dT)-induced IL-1β secretion or flagellin- or poly(dA:dT)-induced CASP1 cleavage in LPS-primed mouse PMs (Figure 4E and Supplemental Figure 4C). CASP1 cleavage following NLRP3 inflammasome activation also markedly decreased in *Isg15* knockdown PMs (Figure 4F). However, *Isg15* knockdown did not affect the activation of NF-κB (Supplemental Figure 4D). Collectively, these data indicated that ISG15 enhanced NLRP3 protein expression and downstream CASP1 activity.

**Figure 5:**
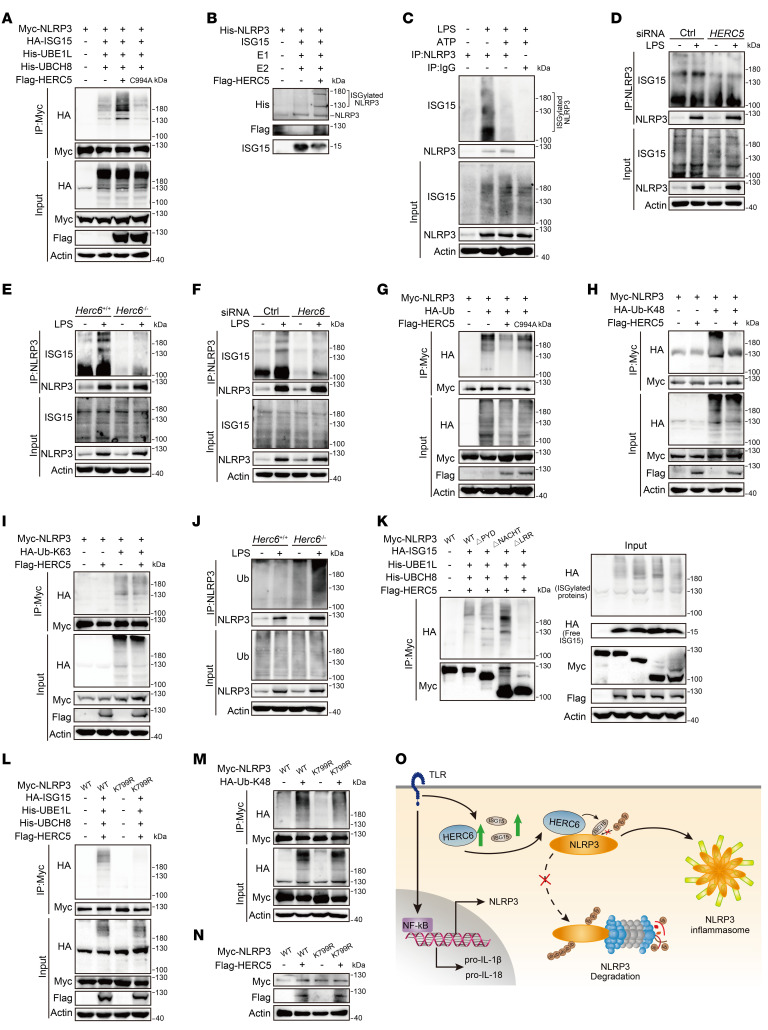
HERCs promote NLRP3 ISGylation. (**A**) Immunoblot analysis of lysates from HEK293T cells transfected with Myc-NLRP3, HA-ISG15, His-UBA7 (His-UBE1L), His-UBE2L6 (His-UBCH8), and Flag-HERC5 WT or C994A for 24 hours, together with MG132 treatment for 4 hours — followed by co-IP with Myc antibody. (**B**) Flag-HERC5 was obtained by in vitro transcription and translation. In vitro ISGylation assay was performed in the presence of ISG15, E1, E2, His-NLRP3, and Flag-HERC5. Immunoblot analysis of the ISGylation of NLRP3 by an anti-His antibody. (**C**) Immunoblot analysis of lysates from mouse PMs, stimulated with LPS for 8 hours or primed with LPS for 7 hours and stimulated with ATP for 1 hour — followed by co-IP with NLRP3 antibody. (**D**) Immunoblot analysis of THP-1 cell lysates transfected with Ctrl or *HERC5* siRNA for 48 hours, primed with LPS for 4 hours, together with MG132 treatment for an additional 4 hours — followed by co-IP with NLRP3 antibody. (**E**, **F**, and **J**) Immunoblot analysis of lysates of mouse PMs from *Herc6*^+/+^ or *Herc6*^–/–^ mice (**E** and **J**) or transfected with Ctrl siRNA or *Herc6* siRNA for 48 hours (**F**), primed with LPS for 4 hours, together with MG132 treatment for an additional 4 hours — followed by co-IP with NLRP3 antibody. (**G**, **H**, and **I**) Immunoblot analysis of lysates from HEK293T cells transfected with Myc-NLRP3, HA-Ub (**G**) or HA-K48–linked ubiquitin (HA-Ub-K48) (**H**) or HA-K63-linked ubiquitin (HA-Ub-K63) (**I**), and Flag-HERC5 WT or C994A for 24 hours, together with MG132 treatment for an additional 4 hours — followed by co-IP with Myc antibody. (**K** and **L**) Immunoblot analysis of lysates from HEK293T cells transfected with Myc-NLRP3 or its truncation mutants (**K**) or mutant K799R (**L**), HA-ISG15, His-UBA7, and His-UBE2L6, and Flag-HERC5 for 24 hours, together with MG132 treatment for 4 hours — followed by co-IP with Myc antibody. (**M**) Immunoblot analysis of lysates from HEK293T cells transfected with Myc-NLRP3 or its mutant K799R, and HA-Ub-K48 for 24 hours, together with MG132 treatment for 4 hours — followed by co-IP with Myc antibody. (**N**) Immunoblot analysis of lysates from *Herc6*^–/–^ MEFs transfected with Myc-NLRP3 or its mutant K799R, and Flag-HERC5 for 24 hours. (**O**) Working model for HERC6 promotion of NLRP3 inflammasome activation. Similar results were obtained from 3 independent experiments.

No interaction between ISG15 and NLRP3 was observed when the respective plasmids were transfected into HEK293T cells (Supplemental Figure 5A). The ISGylation cascade system was constructed in HEK293T cells by cotransfecting the E1 enzyme UBE1L, E2 enzyme UBCH8, and E3 ligase HERC5. NLRP3 ISGylation was enhanced by HERC5 (Figure 5A). However, the HERC5 point mutant (C994A, with substitution of the cysteine residue with alanine at position 994), which lost its E3 ISGylation ligase activity (16), did not enhance NLRP3 ISGylation (Figure 5A), indicating that HERC5 could promote ISGylation of NLRP3 through its E3 ISGylation ligase activity. To determine whether HERC5 directly modified NLRP3, we reconstituted the NLRP3 ISGylation reaction in vitro. Recombinant NLRP3, HERC5, purified E2 enzyme UBCH8, E1 enzyme UBE1L, and ISG15 were prepared. NLRP3 was observed to be ISGylated when ISGylation-related enzymes were present (Figure 5B). Under physiological conditions, NLRP3 was ISGylated following LPS stimulation, whereas ATP and Nig stimulation decreased NLRP3 ISGylation in both mouse PMs and THP-1 cells (Figure 5C and Supplemental Figure 5B). These data indicated that NLRP3 could be ISGylated and suggested dynamic changes in NLRP3 ISGylation during NLRP3 inflammasome activation.

*HERC5* knockdown inhibited NLRP3 ISGylation in LPS-stimulated THP-1 cells (Figure 5D). *Herc6* deficiency and knockdown also attenuated LPS-induced NLRP3 ISGylation in mouse PMs (Figure 5, E and F). As ubiquitination is required for proteasomal degradation of NLRP3, we next examined the effect of HERCs on NLRP3 ubiquitination. HERC5, but not the C994A mutant, inhibited NLRP3 ubiquitination (Figure 5G). In addition, HERC5 selectively inhibited K48-linked, but not K63-linked, ubiquitination of NLRP3 (Figure 5, H and I). Similarly, *Herc6* deficiency enhanced NLRP3 ubiquitination in the mouse PMs (Figure 5J).

Next, we attempted to identify ISGylation sites of NLRP3. We examined the ISGylation of truncated NLRP3 mutants and found that ISGylation of NLRP3 ΔLRR (a mutant in which the LRR domain was deleted) was considerably suppressed (Figure 5K and Supplemental Figure 5C), which suggested that the LRR domain may be required for ISGylation. Given that NLRP3 ISGylation by HERC affects ubiquitination of the protein, we used the UbPred program (http://www.ubpred.org) to predict the possible ubiquitination sites of human NLRP3 and aligned them with the murine NLRP3 protein sequence. Five lysine residues (Lys194, Lys324, Lys430, Lys689, and Lys799) were predicted to be possible ubiquitination sites and were also conserved in both humans and mice (Supplemental Table 1). Among these residues, only Lys799 is located within the LRR domain. Therefore, we replaced the NLRP3 lys799 residue with an arginine (K799R). We found that ISGylation of NLRP3 K799R was weaker than that of WT NLRP3 in HEK293T cells and *Nlrp3*-deficient MEFs (Figure 5L and Supplemental Figure 5D). Meanwhile, total and K48-linked ubiquitination was attenuated in K799R mutants (Figure 5M and Supplemental Figure 5E), suggesting that NLRP3 can be ubiquitinated at K799. Furthermore, HERC5 augmented protein levels of NLRP3, but not the mutant K799R, in *Herc6*-deficient MEFs (Figure 5N). Similarly, *Herc6* deficiency resulted in a reduction in NLRP3, but not mutant K799R, protein levels (Supplemental Figure 5F). We next studied the reported (Lys496 [ref. 17]), Lys689 [ref. 18]) and the predicted (Lys93, Lys324, and Lys696 [ref. 18]) K48-linked ubiquitination sites of NLRP3. We individually replaced each of these 5 NLRP3 lysine residues with arginine (K496R, K93R, K324R, K689R, and K696R). However, all of the aforementioned mutants had the same level of ISGylation as WT (Supplemental Figure 5, G and H). Therefore, HERCs promoted ISGylation of NLRP3 at K799 and thus suppressed its degradation by the proteasome (see the model in Figure 5O).

### Herc6 deficiency ameliorates NLRP3-dependent inflammation in vivo.

**Figure 6:**
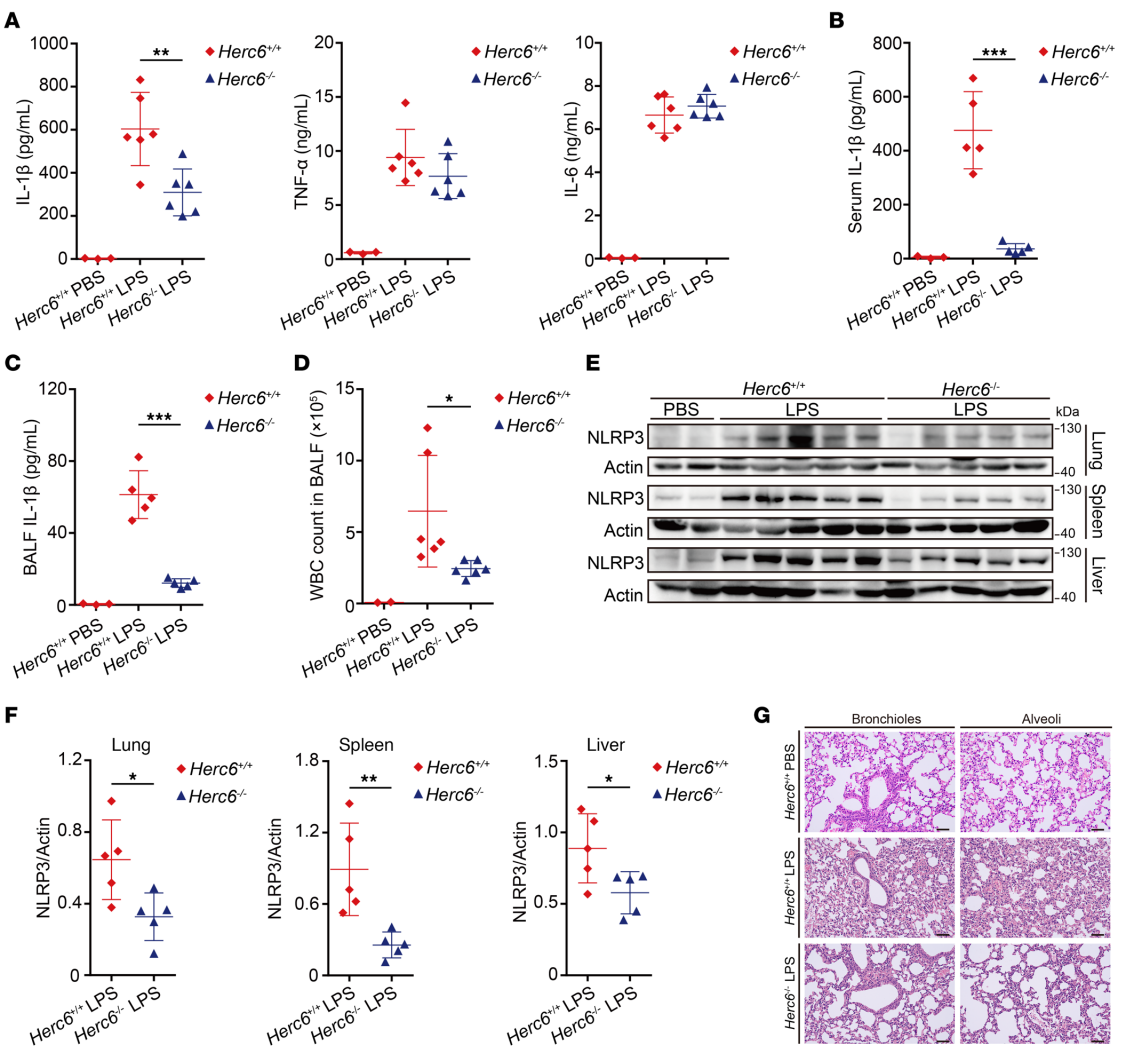
*Herc6* deficiency ameliorates NLRP3-dependent inflammation in vivo. (**A**) ELISA analysis of serum levels of IL-1β, TNF-α, and IL-6 of *Herc6*^+/+^ or *Herc6*^–/–^ mice after i.p. LPS injection for 4 hours (PBS *n* = 3, LPS, *n* = 6 per group; 2-tailed *t* test, *Herc6*^+/+^ vs. *Herc6*^–/–^, **P = 0.005057). (**B** and **C**) ELISA analysis of serum (**B**) or BALF (**C**) IL-1β levels of *Herc6*^+/+^ or *Herc6*^–/–^ mice after i.p. LPS injection for 12 hours (PBS *n* = 3; LPS *n* = 5 per group; 2-tailed *t* test, *Herc6*^+/+^ vs. *Herc6*^–/–^, **B**: ***P = 1.36 × 10^–4^, **C**: ***P = 3.84 × 10^–5^). (**D**) WBC count in BALF of IL-1β of *Herc6*^+/+^ or *Herc6*^–/–^ mice after i.p. LPS injection for 12 hours (PBS *n* = 2, LPS, *n* = 6 per group; 2-tailed *t* test, *Herc6*^+/+^ vs. *Herc6*^–/–^, *P = 0.031741). (**E** and **F**) Immunoblot analysis of lysates from the lung, spleen, and liver of *Herc6*^+/+^ or *Herc6*^–/–^ mice after i.p. LPS injection for 12 hours. NLRP3 expression was quantitated by measuring band intensities using ImageJ software. The values were normalized to actin (PBS *n* = 2, LPS *n* = 5 per group; 2-tailed *t* test, *Herc6*^+/+^ vs. *Herc6*^–/–^, lung: *P = 0.025147, spleen: **P = 0.007801, liver: *P = 0.040026). (**G**) H&E staining of lung tissue sections from *Herc6*^+/+^ or *Herc6*^–/–^ mice after i.p. LPS injection for 12 hours (PBS *n* = 2, LPS *n* = 4 per condition). Scale bars: 10 μm. All data are presented as mean ± SD in **A**–**D** and **F**. Similar results were obtained from 3 independent experiments.

Induction of IL-1β by i.p. injection of LPS is NLRP3 dependent (19). Secretion of IL-1β in serum of *Herc6^–/–^* mice decreased upon LPS challenge, whereas TNF-α and IL-6 levels remained unchanged (Figure 6A). Acute respiratory distress syndrome (ARDS) is a serious inflammatory disorder and the most common immediate cause of death due to infection, such as COVID-19 (20, 21). Therefore, we constructed a mouse ARDS model using i.p. injection of LPS to further investigate the physiological and pathological relevance of HERC6 in inflammation in vivo. After i.p. injection of LPS, the mice showed respiratory weakness and slow movement, and began to die after 24 hours. IL-1β secretion in *Herc6^–/–^* mouse serum decreased upon LPS challenge, whereas TNF-α and IL-6 levels remained unchanged (Figure 6B and Supplemental Figure 6A). The secretion of IL-1β, TNF-α, and IL-6 in bronchoalveolar lavage fluid (BALF) of *Herc6^–/–^* mice decreased after the LPS challenge (Figure 6C and Supplemental Figure 6B). The number of WBCs in the BALF of *Herc6^–/–^* mice was lower than that in WT mice (Figure 6D). NLRP3 protein expression in the lung, spleen, and liver of LPS-challenged *Herc6^–/–^* mice decreased (Figure 6, E and F), whereas *Nlrp3* and *Il1b* mRNA expression remained unchanged (Supplemental Figure 6, C–E). Interestingly, the bronchioles and alveoli of LPS-challenged *Herc6^–/–^* mice exhibited less tissue damage and inflammatory cell infiltration (Figure 6G). These results demonstrated that *Herc6* deficiency ameliorated NLRP3-dependent inflammation and ARDS in vivo.

### Herc6 deficiency attenuates viral infection–caused inflammation.

**Figure 7:**
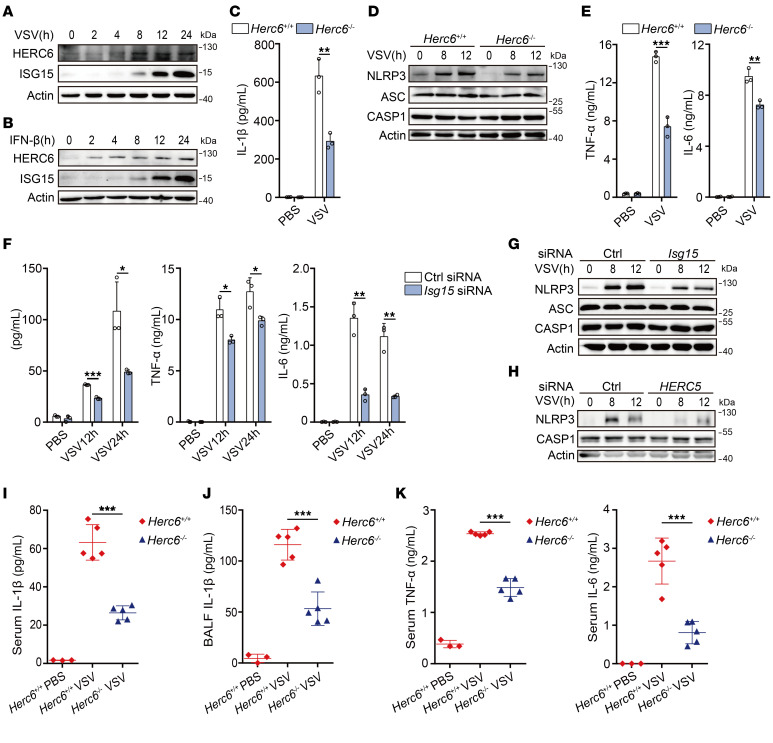
HERC6 promotes VSV-induced inflammation. (**A** and **B**) Immunoblot analysis of HERC6 and ISG15 expression in VSV-infected (**A**) or mIFN-β–stimulated (**B**) mouse PMs. (**C**) ELISA analysis of IL-1β in supernatants of PMs from *Herc6*^+/+^ or *Herc6*^–/–^ mice after infection with VSV for 24 hours (2-tailed *t* test, *Herc6*^+/+^ vs. *Herc6*^–/–^, **P = 0.003646). (**D**) Immunoblot analysis of PMs from *Herc6*^+/+^ or *Herc6*^–/–^ mice after VSV infection. (**E**) ELISA analysis of TNF-α and IL-6 in supernatants of PMs from *Herc6*^+/+^ or *Herc6*^–/–^ mice after VSV infection for 24 hours (2-tailed *t* test, *Herc6*^+/+^ vs. *Herc6*^–/–^, ***P = 0.000399, **P = 0.004338). (**F**) ELISA analysis of IL-1β, TNF-α, and IL-6 in supernatants from mouse PMs transfected with Ctrl or *Isg15* siRNA for 48 hours — following VSV infection for 12 or 24 hours (2-tailed *t* test, Ctrl vs. *Isg15* siRNA, IL-1β: ***P = 0.000059, *P = 0.023051; TNF-α: *P = 0.011107 [12 h], *P = 0.026233 [24h]; IL-6: **P = 0.001473 [12h], **P = 0.001505 [24h]**.** (**G**) Immunoblot analysis of mouse PMs transfected with Ctrl or *Isg15* siRNA for 48 hours, following VSV infection. (**H**) Immunoblot analysis of THP-1 cells transfected with Ctrl or *HERC5* siRNA for 48 hours, following VSV infection. (**I**–**K**) ELISA analysis IL-1β in serum (**I**) and BALF (**J**), or TNF-α and IL-6 in serum (**K**) of *Herc6*^+/+^ or *Herc6*^–/–^ mice after infection with VSV by i.p. injection for 12 hours (PBS *n* = 3, VSV *n* = 5 per group; 2-tailed *t* test, *Herc6*^+/+^ vs. *Herc6*^–/–^, **I**: ***P = 3.44 × 10^–5^, **J**: ***P = 0.000233, **K**: TNF-α ***P = 9.78 × 10^–7^, IL-6 ***P = 0.000241). All data are presented as mean ± SD in **C**, **E**, **F**, and **I**–**K**. Similar results were obtained from 3 independent experiments.

We observed that vesicular stomatitis virus (VSV, a type of RNA virus) infection and IFN-β stimulation induced HERC6 and ISG15 expression in mouse PMs (Figure 7, A and B, and Supplemental Figure 7, A and B). *Herc6* deficiency and knockdown markedly inhibited VSV infection–induced IL-1β secretion and NLRP3 protein expression (Figure 7, C and D, and Supplemental Figure 7, C and D). Notably, *Herc6* deficiency and knockdown also attenuated TNF-α and IL-6 secretion (Figure 7E and Supplemental Figure 7E) and mRNA expression of *Nlrp3* and *Il1b* (Supplemental Figure 7, F and G) following VSV infection. Similar results were observed in *Isg15*-knockdown mouse PMs (Figure 7, F and G, and Supplemental Figure 7G). Consistent with these findings, *HERC5* knockdown inhibited NLRP3 protein expression in VSV-infected THP-1 cells (Figure 7H).

The regulatory effects of HERC6 on the NLRP3 inflammasome and the role of VSV infection on inflammation were then investigated in vivo. In the BALF of VSV-infected *Herc6^–/–^* mice, IL-1β secretion was much lower than that in the BALF of WT mice (Figure 7I). In addition, VSV infection–induced secretion of IL-1β, TNF-α, and IL-6 in the serum of *Herc6*-deficient mice was markedly decreased (Figure 7, J and K). Taken together, these data indicate that *Herc6* deficiency attenuated viral infection–induced inflammasome activation by inhibiting NLRP3 expression and therefore ameliorated viral infection–induced hyperinflammation in vivo.

## Discussion

Posttranslational NLRP3 modification induces inflammasome activation and determines the inflammation intensity (1). In the resting state, NLRP3 is held in an inactive conformation at least partly by PTMs. Dynamic changes in PTMs, such as phosphorylation and ubiquitination, upregulate NLRP3 protein levels and license NLRP3 to rapidly respond to stimuli. Cellular NLRP3 levels are considered rate-limiting for canonical NLRP3 inflammasome activation (22). Several E3 ubiquitin ligases — including tripartite motif–containing protein 31 (TRIM31) (23), F-box/LRR-repeat protein 2 (FBXL2) (24), membrane-associated RING finger protein 7 (MARCH7) (25), and casitas B–lineage lymphoma protein-b (Cbl-b) (17) — induce K48-linked ubiquitination and protein degradation of NLRP3, which limits cellular NLRP3 levels and inflammasome assembly. BRCA1/BRCA2-containing complex subunit 3 (BRCC3) deubiquitinates NLRP3 and is required for NLRP3 oligomerization and activation (26). The ubiquitin-specific peptidase 1 (USP1)/USP1-associated factor 1 (UAF1) deubiquitinase complex removes K48-linked ubiquitination of NLRP3 and stabilizes NLRP3 protein levels to facilitate inflammasome activation (27). The phosphorylation of NLRP3 at Ser198 (in human NLRP3; Ser194 in mouse NLRP3) induced by JNK1 promotes BRCC3 binding and subsequent NLRP3 deubiquitination, resulting in the enhancement of NLRP3 self-association and activation (28). However, phosphorylation of NLRP3 induced by the kinases Lyn (29), PKA (30), and PKB (AKT) (31) inhibits NLRP3 activation by promoting its ubiquitination and degradation. In addition to phosphorylation, a small ubiquitin-like modifier (SUMO) modification (called SUMOylation) mediated by the E3 SUMO ligase TRIM28 controls NLRP3 inflammasome activation by inhibiting ubiquitination and degradation of NLRP3 (32). In the present study, ISGylation of NLRP3 by HERCs (HERC5 in humans and HERC6 in mice) following TLR priming was demonstrated. In contrast, NLRP3 ISGylation was decreased following ATP or Nig stimulation. These results suggest that ISGylation of NLRP3 is favorable in the priming phase because it stabilizes NLRP3 expression. The potential roles of NLRP3 deISGylation in inflammasome activation need to be further investigated. HERC-mediated NLRP3 ISGylation stabilizes NLRP3 by inhibiting its ubiquitination and proteasomal degradation, thereby activating the NLRP3 inflammasome. ISGylation is a type of PTM in which ISG15 covalently binds to lysine (K) residues of target proteins. Therefore, ISG15 modification of NLRP3 may block ubiquitin conjugation by competing with the same K residues of NLRP3 (8). Further analysis of the ISGylation and ubiquitination sites of NLRP3 will help elucidate dynamic changes in PTMs of NLRP3 and their reciprocal regulation in inflammasome activation.

Type I IFNs and inflammatory responses are crucial for host defense against pathogens, especially viral infections. However, unbalanced inflammation and type I IFN responses can lead to immunopathology. For example, low levels of type I IFNs and a moderate ISG response coupled with excessive proinflammatory cytokines are the defining and driving features of COVID-19 (33). Thus, mechanisms that ensure the beneficial production of type I IFNs and proinflammatory cytokines (such as IL-1β, TNF-α, and IL-6) are of particular importance. Emerging evidence has documented the reciprocal regulation of type I IFNs and inflammatory responses and highlights the importance of their crosstalk in maintaining immune homeostasis. IL-1R1 promotes the TLR9-dependent type I IFNs response by enhancing K63-linked polyubiquitylation of TNF receptor–associated factor 3 (TRAF3) (34). Type I IFNs induce IL-10 expression in a STAT1-dependent manner to reduce the abundance of pro–IL-1 and inflammasome activation (35). Notably, type I IFNs can promote the transcription of *NLRP3* and priming of the NLRP3 inflammasome by binding to IFNAR and activating the JAK/STAT axis (36, 37). These reports highlight the complexity of type I IFNs in inflammasome control. Although the effects of type I IFNs on NLRP3 inflammasome activation remain controversial, the enhancement of inflammation by some ISGs has been documented. Retinoic acid–inducible gene 1 protein (RIG-I), a key pattern recognition receptor (PRR) induced by type I IFNs, detects RNA viruses to initiate the type I IFNs responses and binds ASC to enhance inflammasome activation (38). Guanylate-binding protein 5 (GBP5) promotes NLRP3 oligomerization and NLRP3 inflammasome activation (39). ISG15 acts as a cytokine that enhances IFN-γ and exacerbates SARS-CoV-2 infection–triggered inflammation (11, 13, 40). In this study, ISG15 was shown to facilitate NLRP3 inflammasome activation and enhance inflammation in an ISGylation-dependent manner. Type I IFNs induced the expression of ISG15 and HERCs, which then enhanced inflammasome activation by promoting ISGylation and NLRP3 expression. These results illustrate the mechanisms by which type I IFNs responses control inflammasome activation and viral infection–induced excessive NLRP3 inflammasome activation. VSV infection has more complex effects on innate signaling pathways, including activation of the NLRP3 inflammasome and RIG-I-like receptor (RLR) pathway. It has been reported that several key molecules of the type I IFN signaling pathway, such as RIG-I, could be ISGylated (41), which may explain why *Herc6* deficiency not only suppressed NLRP3 protein expression, but also reduced TNF-α and IL-6 expression, during VSV infection.

In summary, ISGylation has been identified as a PTM of NLRP3 that induces inflammasome activation. The E3 ISGylation enzymes HERCs (HERC5 in humans and HERC6 in mice) mediated ISGylation of NLRP3, inhibited K48-linked ubiquitination and proteasomal degradation of NLRP3, and thus enhanced inflammasome activation. *Herc6* deficiency ameliorated NLRP3-dependent inflammation and ARDS (a direct cause death in patients with COVID-19) in vivo. Aberrant NLRP3 activation is central to deleterious inflammation in severe viral infections, nervous system diseases, metabolic diseases, autoimmune diseases, and cancer (42). Therefore, HERCs are promising targets for preventing and treating diseases caused by excessive NLRP3 activation.

## Methods

### Mice.

*Herc6*-deficient mice on a C57BL/6 background were generated by Cyagen Biosciences Inc. by CRISPR/Cas9-mediated genome editing. *Ifnar*-deficient (stock 028288) and *Nlrp3*-deficient (stock 021302) C57BL/6 mice were obtained from Jackson Laboratory. C57BL/6 mice were purchased from the Beijing Vital River Laboratory Animal Technology Co. Mice were kept in an specific pathogen–free–level laboratory animal room with 12-hour light/12-hour dark alternating periods, with temperature 18°C–22°C and humidity 45%–65%.

### Cell culture.

To obtain mouse primary PMs, we injected C57BL/6 mice (6–8 weeks old) i.p. with 3% Brewer thioglycollate broth. After 3 days, the peritoneal exudate cells were harvested and incubated. Two hours after this, nonadherent cells were removed, and adherent monolayer cells were used as PMs. THP-1 and HEK293T cells were obtained from ATCC. PMA-activated THP-1 cells were used as human macrophages. MEFs were obtained as described previously (23). The cells were cultured at 37°C in 5% CO_2_ in medium supplemented with 10% FCS (Invitrogen). PMs, MEFs, and HEK293T cells were cultured in DMEM. THP-1 cells were cultured in RPMI medium.

### Reagents and antibodies.

ATP (A1852), Nig sodium salt (N7143), Z-Leu-Leu-Leu-al (MG132, C2211), LPS (*E*. *coli*, O111:B4, L4130), anti-Myc (M4439, 1:5,000), anti-HA (H3663, 1:1,000), and anti-Flag M2 (F1804, 1:1,000) were obtained from MilliporeSigma; CHX (A8244) was from APExBIO Technology; poly(dA:dT) (tlrl-patn), flagellin (tlrl-epstfla), and MSU Crystals (tlrl-msu-25) were from Invivogen; recombinant human ISG15 protein (UL-601), recombinant human ISG15 E1/UBE1L protein (E-309), recombinant human UBCH8/UBE2L6 protein (E2-644-100), and 10× ubiquitin conjugation reaction buffer were from R&D Systems; mouse IFNB1/IFN-beta/Interferon beta Protein (50708-MCCH) was from Sino Biological; recombinant murine IL-1β (211-11B) was from PeproTech China; anti-mouse IgG (7076, 1:5,000), anti–p-IκBα (9246, 1:1,000), anti-IκBα (4814, 1:1,000), anti-AIM2 (13095S, 1:1,000), and anti-ISG15 (2743, 1:1,000) were from Cell Signaling Technology; anti–caspase-1 p20 (AG-20B-0042, 1:1,000), anti-NLRP3 (AG-20B-0014, 1:1,000), and anti-ASC (AG-25B-0006, 1:1,000) were from AdipoGen; anti-Ub (sc-8017, 1:1,000) and protein G agarose (sc-2002) used for IP were from Santa Cruz Biotechnology Inc.; anti-NLRP3 (ab263899, 1:1,000), anti-NEK7 (ab133514, 1:1,000), anti–caspase-1 (ab179515, 1:1,000), anti-HERC6 (ab22553, 1:1,000), and anti–IL-1β (ab234437, 1:1,000) were from Abcam; anti-NLRP3 (19771-1-AP, 1:1,000), anti-His (66005-1-Ig, 1:1,000), anti-HERC5 (22692-1-AP, 1:1,000), HRP-conjugated goat anti-rabbit IgG (H+L) (SA00001-2, 1:5,000), anti–β-actin (66009-1-Ig, 1:2,000), HRP-conjugated IgG Fraction Monoclonal Mouse Anti-Rabbit IgG, Light Chain Specific (SA00001-7L, 1:5,000), and HRP-conjugated Recombinant Rabbit Anti-Mouse IgG, Kappa Light Chain (SA00001-19, 1:5,000) were from Proteintech; goat anti-mouse IgG (H+L) cross-adsorbed secondary antibody and alum (catalog 77161) were from Thermo Fisher Scientific; anti-Myc (N7143, 1:1,000) and purified recombinant NLRP3 (WX030FB0) were from Origene.

### Stimulants and viruses.

The concentrations of agonists or stimulants were as follows: LPS, 200 ng/mL for mouse PMs; LPS, 1 μg/mL for THP-1 cells and MEFs; ATP, 2.5 mM; Nig, 10 μM; MSU, 250 μg/mL; alum, 2 mg/mL; flagellin, 500 ng/mL; MG132, 10 μM. Poly(dA:dT) was transfected into the macrophages at a final concentration of 200 ng/mL. VSV-GFP was obtained from X. Cao (Second Military Medical University, Shanghai, China).

### Plasmid construction and transfection.

Expression plasmids for FLAG-tagged human HERC5, FLAG-tagged human HERC6, Flag-tagged mouse HERC6, Myc-tagged mouse NLRP3, His-tagged human UBE1L, and His-tagged human UBCH8 were purchased from Changsha Youze Biotechnology Co. FLAG-tagged human ARIH1 and FLAG-tagged human TRIM25 were purchased from WZ Biosciences Inc. Human HERC5 C994A and NLRP3 mutant K93R, K324R, K430R, K496R, K689R, K696R, and K799R were generated using a KOD-Plus-Mutagenesis Kit (Toyobo). The HA-tagged human ISG15 expression plasmid was constructed in our laboratory. Expression plasmids for Myc-tagged human NLRP3, Myc-tagged human ASC, HA-tagged human caspase-1, HA-tagged human Ub WT, mutant K48, and mutant K63 have been described previously. All the constructs were confirmed by DNA sequencing. Plasmids were transiently transfected into HEK293T cells using Lipofectamine 2000 reagent (Invitrogen) and into MEFs using X-tremeGENE HP DNA transfection reagent (MilliporeSigma, 6366244001) according to the manufacturer’s instructions.

### RNA interference assay.

siRNAs were synthesized using the following primers: mouse *Herc6*, 5′-GCUUUCCAUGACUUAACUUTT-3′, mouse *Isg15*, 5′-CCAUGACGGUGUGUCAGAACUUTT-3′; human *HERC5*, 5′-GACGCCGAAAUGCAUUAAATT-3′; negative control, 5′-UUCUCCGAACGUGUCACGU-3′. These siRNA duplexes were transfected into mouse PMs or THP-1 cells using INTERFERin reagent (Polyplus Transfection) according to the manufacturer’s instructions.

### ELISA assay.

Concentrations of mouse IL-1β (catalog 1210123), TNF-α (catalog 1217202), and IL-6 (catalog 1210603) were measured using ELISA kits (Dakewe Biotech Company) according to the manufacturer’s instructions.

### RNA quantitation.

RNA from PMs or THP-1 cells was extracted using the RNAfast200 RNA Extraction kit (Fastagen Biotech), according to the manufacturer’s instructions. Total RNA from tissues was extracted with the TRIzol reagent according to the manufacturer’s instructions (Invitrogen). Quantitative real-time PCR (RT-PCR) analysis was performed using the Applied Biosystems StepOnePlus Real-Time PCR System and SYBR RT-PCR kits (Roche) (43). The specific primers used for real-time PCR assays are listed in Supplemental Table 2. The data were normalized to m*Actb* or h*ACTIN* expression in each sample.

### Co-IP.

For co-IP, whole-cell lysates were lysed in IP buffer containing 1.0% (vol/vol) Nonidet P 40, 50 mM Tris-HCl, pH 7.4, 50 mM EDTA, 150 mM NaCl, and a protease inhibitor cocktail (MilliporeSigma). After 15–20 minutes of centrifugation at 12,000*g* at 4°C, protein concentrations in the lysates were measured with a bicinchoninic acid assay (Pierce), and the supernatants were collected and incubated overnight with protein G Plus-Agarose immunoprecipitation reagent together with specific antibodies. The beads were washed 5 times with IP buffer. Immunoprecipitates were eluted by boiling in 1% (wt/vol) SDS sample buffer (44). For co-IP of ubiquitination and ISGylation, whole-cell lysates were lysed in 40 μL SDS lysis buffer containing 5.0% (vol/vol) glycerol, 50 mM Tris-HCl, pH 6.8, 40 mM DTT, 1.0% (wt/vol) SDS, and a protease inhibitor cocktail. After 30 minutes of centrifugation at 12,000*g* at 4°C, lysates were heated to 100°C for 10 minutes. Protein concentrations in the lysates were measured with a bicinchoninic acid assay and diluted 1:10 with IP buffer, and supernatants were collected and incubated with protein G Plus-Agarose IP reagent together with specific antibodies overnight. The beads were washed 5 times with IP buffer. The immunoprecipitates were eluted by boiling in 1% (wt/vol) SDS sample buffer.

### Immunoblot analysis.

For immunoblot analysis, cells were lysed with RIPA buffer (Pierce) supplemented with a protease inhibitor cocktail. After 15 minutes of centrifugation at 12,000*g* at 4°C, protein concentrations in the lysates were measured using a bicinchoninic acid assay. Equal amounts of lysates were separated by SDS-PAGE and transferred onto nitrocellulose membranes for immunoblot analysis. Cell culture supernatants were harvested and concentrated for immunoblotting with Amicon Ultra 10 K (MilliporeSigma). NLRP3 expression in chase assays was quantitated by measuring band intensities using ImageJ software (NIH). Values were normalized to those of actin.

### In vitro ISGylation assay.

Flag-HERC5 proteins were expressed using the TNT Quick Coupled Transcription/Translation System (Promega). In vitro ISGylation assays involve mixing the corresponding proteins, adding ATP and buffers, and then incubating at 37°C for 1 hour. Equal amounts of lysates were separated by SDS-PAGE and transferred onto nitrocellulose membranes for immunoblot analysis.

### LPS-induced sepsis model in mice.

For in vivo experiments, mice (male, 8 weeks old) were i.p. injected with 25 mg/kg LPS or PBS. After 4 hours, the mice were euthanized, and blood was collected to measure levels of the serum cytokines IL-1β, TNF-α, and IL-6 by ELISA. This method was adapted and modified from that of Sun et al. (45).

### LPS-induced ARDS model in mice.

For in vivo experiments, mice (male, 8 weeks old) were i.p. injected with 10 mg/kg LPS or PBS for 12 hours. Peripheral blood serum and BALF were collected to measure the cytokines IL-1β, TNF-α, and IL-6 by ELISA. WBCs in BALF were counted. Proteins and RNA in the lungs, spleen, and liver were extracted for immunoblot and RT-PCR analyses. Lung tissue from mice was fixed in 4% paraformaldehyde, embedded in paraffin, sectioned, stained with hematoxylin, and examined by light microscopy for histological changes. This method was adapted and modified from that of Xian et al. (46).

### Viral pathogenesis in mice.

For in vivo experiments, mice (male, 8 weeks old) were i.p. infected with VSV (5 × 10^8^ PFU/mouse). After 16 hours, the mice were euthanized, and peripheral blood serum and BALF were collected to measure cytokines such as IL-1β, TNF-α, and IL-6 by ELISA. This method was adapted and modified from that of Wang et al. (47) and Allen et al. (48).

### Statistics.

Significant differences between groups were determined by a 2-tailed Student’s *t* test using GraphPad Prism 6.0. Statistical significance was set at *P* < 0.05.

### Study approval.

All animal experiments were performed in accordance with the NIH *Guide for the Care and Use of Laboratory Animals* (National Academies Press, 2011), with the approval of the Scientific Investigation Board of the School of Basic Medical Science.

### Data availability.

All data supporting the findings of this study are available within the article and supplemental material, with all data points in graphs reported in the Supporting Data Values file; and from the corresponding author upon reasonable request. See complete unedited blots in the supplemental material.

## Author contributions

WZ designed and supervised the research; YQ, XM, MW, WL, RX, JC, HS, YF, MJ and CZ performed the experiments; JL and CG provided expertise and advice; and YQ and WZ analyzed the data and wrote the manuscript.

## Supplementary Material

Supplemental data

Supporting data values

## Version 1 08/31/2023

In-Press Preview

## Version 2 10/16/2023

Electronic publication
