# Supplementary material for: Posttranslational ISGylation of NLRP3 by HERC enzymes facilitates inflammasome activation in models of inflammation
Source: J Clin Invest. 2023 Oct 16;133(20):e161935. doi: 10.1172/JCI161935 (PMC10575725; doi:10.1172/JCI161935)
Supplement: Supplemental data [file jci-133-161935-s173.pdf]

Supplementary Information for

Posttranslational ISGylation of NLRP3 by HERCs enzymes facilitates inflammasome activation in models of inflammation

Ying Qin, Xintong Meng, Mengge Wang, Wenbo Liang, Rong Xu, Jingchunyu Chen, Hui Song, Yue Fu, Jingxin Li, Chengjiang Gao, Mutian Jia, Chunyuan Zhao, and Wei Zhao *

Key Laboratory for Experimental Teratology of the Chinese Ministry of Education and Key Laboratory of Infection and Immunity of Shandong Province, School of Basic Medical Science, Qilu Hospital, Cheeloo College of Medicine, Shandong University, Jinan, Shandong, China

*Correspondence: wzhao@sdu.edu.cn (W.Z.)

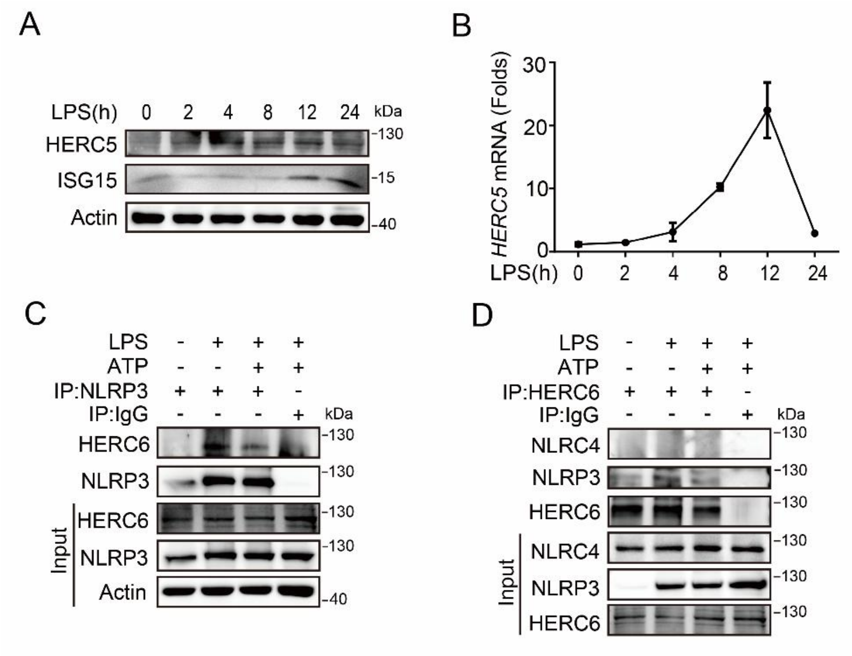

Supplementary Figure 1. LPS-induced HERC5 expression and its interaction with NLRP3

(**A**) Immunoblot analysis of HERC5 and ISG15 expression in LPS-stimulated THP-1 cells.

(**B**) RT-PCR analysis of *HERC5* mRNA in LPS-stimulated THP-1 cells. All data are represented as the mean ± SD.

(**C**) Co-IP analysis of the endogenous association between HERC6 and NLRP3 in LPS-stimulated or LPS-primed and ATP-activated mouse PMs.

(**D**) Co-IP analysis of the endogenous association between HERC6 and NLRC4 or NLRP3 in LPS-stimulated or LPS-primed and ATP-activated mouse PMs.

Similar results were obtained from three independent experiments.

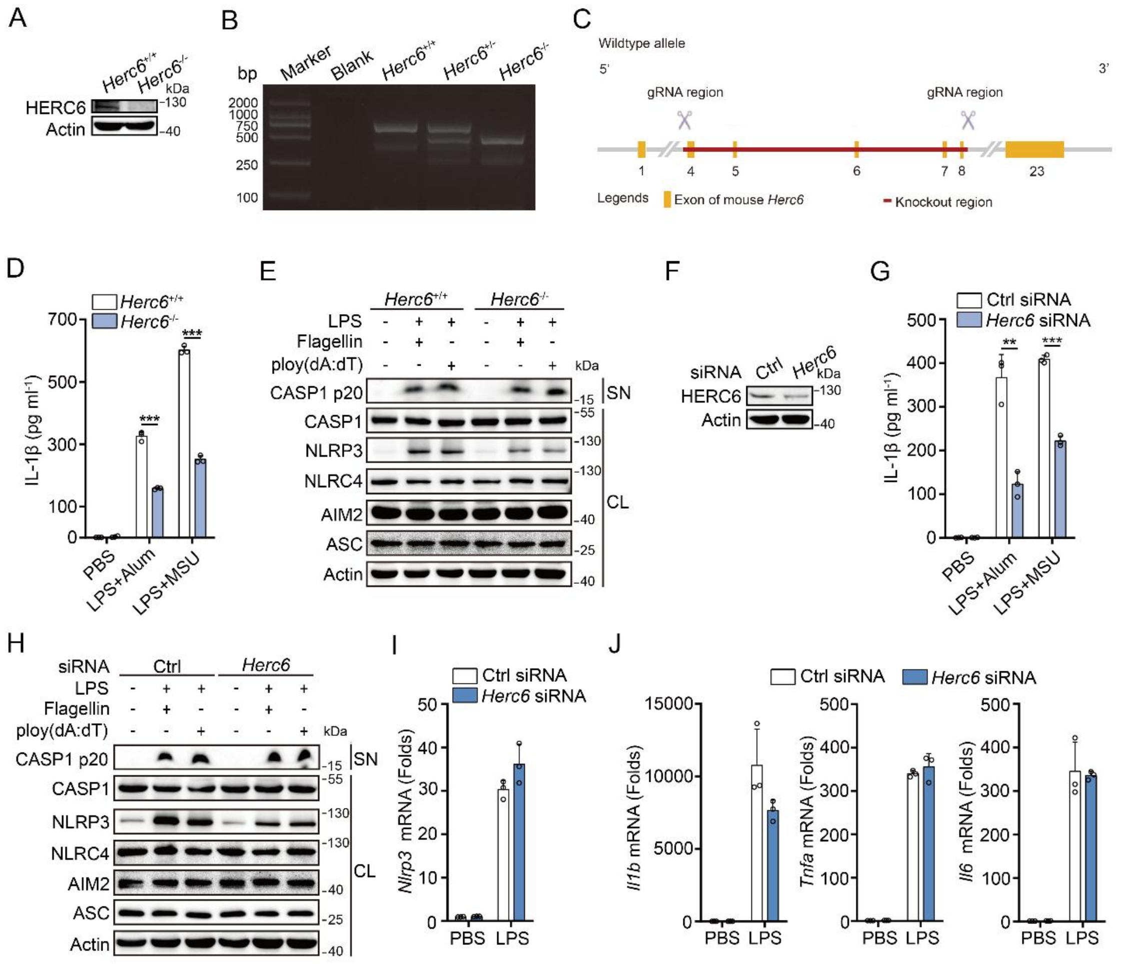

Supplementary Figure 2. ***Herc6*** **knockdown did not affect the priming of NLRP3 inflammasome**

(**A**) Immunoblot analysis of HERC6 expression in PMs from *Herc6*$^{+/+}$ or *Herc6*$^{-/-}$ mice.

(**B**) PCR analysis of *Herc6* deletion in genomes of *Herc6*$^{+/+}$, *Herc6*$^{+/-}$ or *Herc6*$^{-/-}$ mice. *Herc6*$^{+/+}$: 716bp; *Herc6*$^{+/-}$: 716bp and 510bp; *Herc6*$^{-/-}$: 510bp.

(**C**) Schematic representation of mouse *Herc6* gene knockout, provided by Cyagen Biosciences Inc. (Guangzhou, China).

(**D**) ELISA analysis of IL-1β in supernatants of PMs from *Herc6*$^{+/+}$ or *Herc6*$^{-/-}$ mice following priming with LPS for 2 h and subsequent stimulation with Alum or MSU for 6 h (A. two-tailed t-test *Herc6*$^{+/+}$ vs. *Herc6*$^{-/-}$, ***p = 4.67×10^{-5}, 3.88×10^{-6}).

(**E**) Immunoblot analysis of SN and CL of mouse PMs from *Herc6*$^{+/+}$ or *Herc6*$^{-/-}$ mice, following LPS priming for 7h and subsequent Flagellin or poly(dA:dT) stimulation for 1h.

(**F**) Immunoblot analysis of HERC6 expression in mouse PMs transfected with Ctrl or *Herc6* siRNA for 48h.

(**G**) ELISA analysis of IL-1β in supernatants from mouse PMs transfected with Ctrl siRNA or *Herc6* siRNA for 48 h, followed by priming with LPS for 2 h and subsequent stimulation with Alum or MSU for 6 h (two-tailed *t*-test Ctrl siRNA vs. *Herc6* siRNA, **p = 0.002213, ***p = 2.81×10^{-5}).

(**H**) Immunoblot analysis of SN and CL of mouse PMs transfected with Ctrl siRNA or *Herc6* siRNA for 48 h, followed by LPS priming for 7h and subsequent Flagellin or poly(dA:dT) stimulation for 1h.

(**I** and **J**) RT-PCR analysis of *Nlrp3* or *Il1b*, *Tnfa,* and *Il6* mRNA in LPS-stimulated mouse PMs transfected with Ctrl or *Herc6* siRNA.

All data are represented as mean ± SD in D, G, I, and J. Similar results were obtained from three independent experiments.

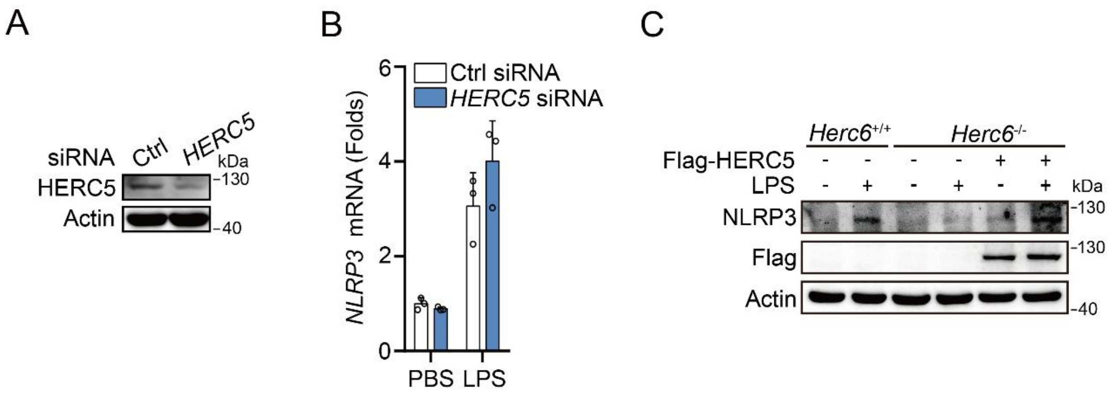

Supplementary Figure 3. *HERC5* knockdown did not affect *NLRP3* mRNA expression

(**A**) Immunoblot analysis of HERC5 expression in THP-1 cells transfected with Ctrl or *HERC5* siRNA for 48h.

(**B**) RT-PCR analysis of *NLRP3* in THP-1 cells transfected with Ctrl or *HERC5* siRNA for 48h, following LPS stimulation for 2h. All data are represented as mean ± SD.

(**C**) Immunoblot analysis of lysates from *Herc6*$^{+/+}$ or *Herc6*$^{-/-}$ MEFs transfected with Flag-hHERC5 or empty vector plasmid, following LPS stimulation for 8h.

Similar results were obtained from three independent experiments.

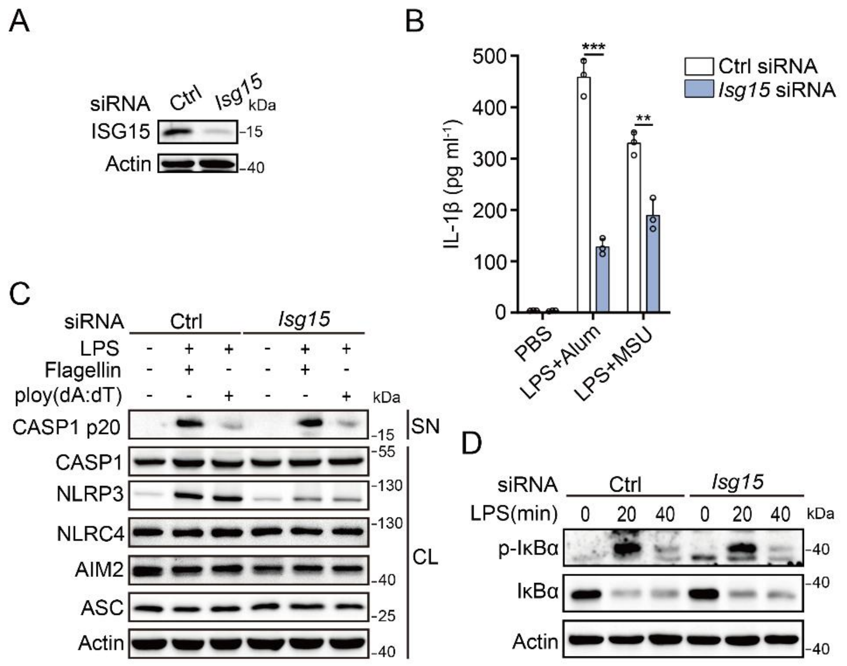

Supplementary Figure 4. ***Isg15*** **knockdown did not affect NF-κB expression**

(**A**) Immunoblot analysis of ISG15 expression in mouse PMs transfected with Ctrl or *Isg15* siRNA for 48h.

(**B**) ELISA analysis of IL-1β in supernatants from mouse PMs transfected with Ctrl or *Isg15* siRNA for 48 h, followed by priming with LPS for 2 h and subsequent stimulation with Alum or MSU for 6 h (two-tailed t-test Ctrl siRNA vs *Isg15* siRNA, ***p = 0.000117, **p = 0.003116).

(**C**) Immunoblot analysis of SN and CL of mouse PMs transfected with Ctrl siRNA or *Isg15* siRNA for 48 h, followed by LPS priming for 7h and subsequent Flagellin or poly(dA:dT) stimulation for 1h.

(**D**) Immunoblot analysis of p-IκBα and IκBα in LPS-stimulated mouse PMs transfected with Ctrl or *Isg15* siRNA for 48h.

Similar results were obtained from three independent experiments.

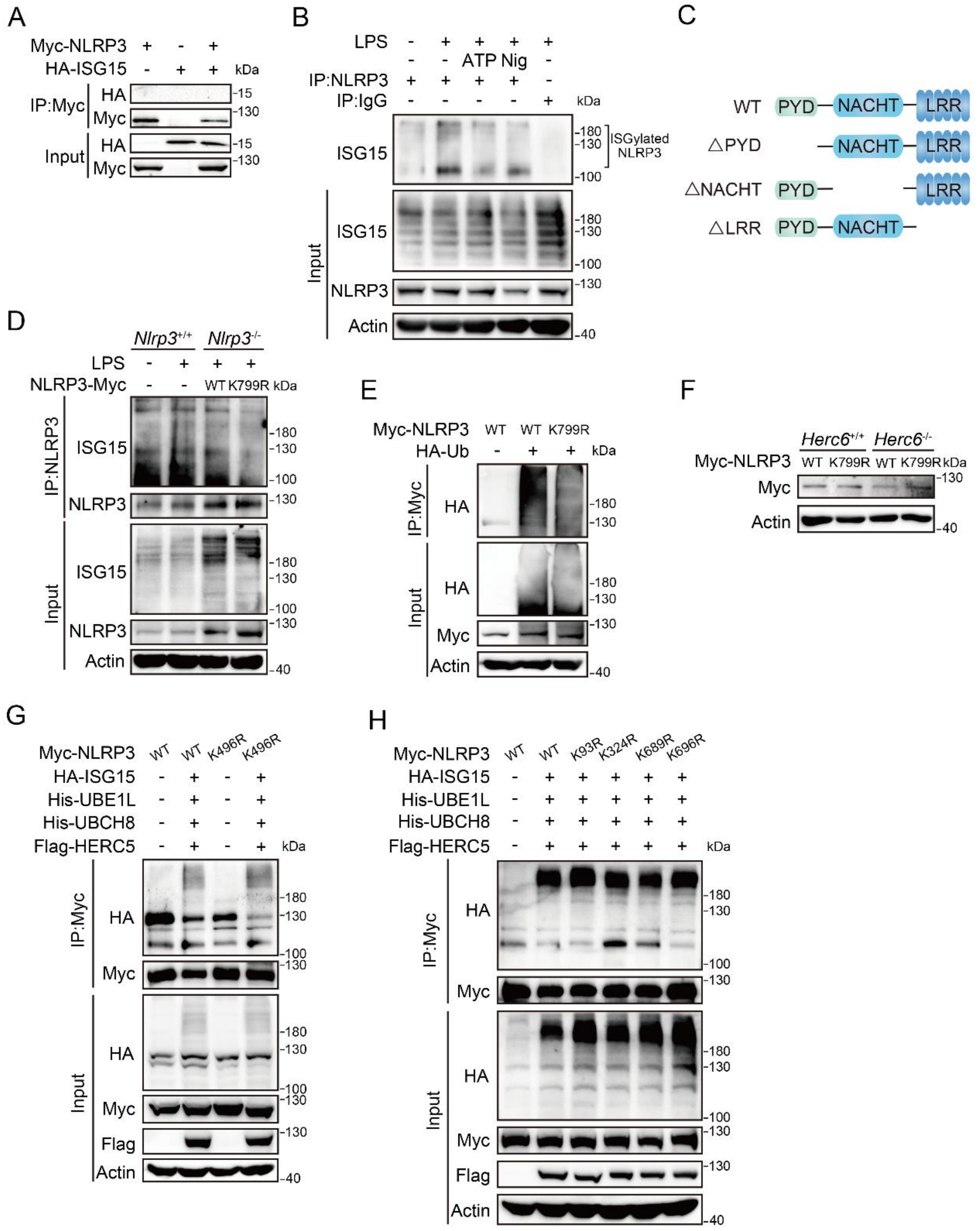

Supplementary Figure 5. NLRP3 was ISGylated in LPS-stimulated THP1 cells

(**A**) Immunoblot analysis of lysates from HEK293T cells transfected with Myc-NLRP3 and HA-ISG15 for 24h followed by co-IP with Myc antibody.

(**B**) Immunoblot analysis of lysates from LPS-stimulated or -primed and ATP- or

Nig-activated THP-1 cells followed by co-IP with NLRP3 antibody.

(**C**) Schematic diagram of NLRP3 and its truncation mutants.

(**D**) Immunoblot analysis of lysates from *Nlrp3*$^{+/+}$ or *Nlrp3*$^{-/-}$ MEFs transfected with Myc-NLRP3 or its mutant K799R for 24 h, LPS stimulation for 4h, together with MG132 treatment for 4 h and followed by co-IP with NLRP3 antibody.

(**E**) Immunoblot analysis of lysates from HEK293T cells transfected with Myc-NLRP3 or its mutant K799R, and HA-Ub for 24 h, together with MG132 treatment for 4 h and followed by co-IP with Myc antibody.

(**F**) Immunoblot analysis of lysates from *Herc6*$^{+/+}$ or *Herc6*$^{-/-}$ MEFs transfected with Myc-NLRP3 or its mutant K799R for 24 h.

(**G** and **H**) Immunoblot analysis of lysates from HEK293T cells transfected with Myc-NLRP3 or its mutant K496R (**G**) or K93R, K324R, K689R and K696R (**H**), HA-ISG15, His-UBA7, and His-UBE2L6, and Flag-HERC5 for 24 h, together with MG132 treatment for 4 h and followed by co-IP with Myc antibody.

Similar results were obtained from three independent experiments.

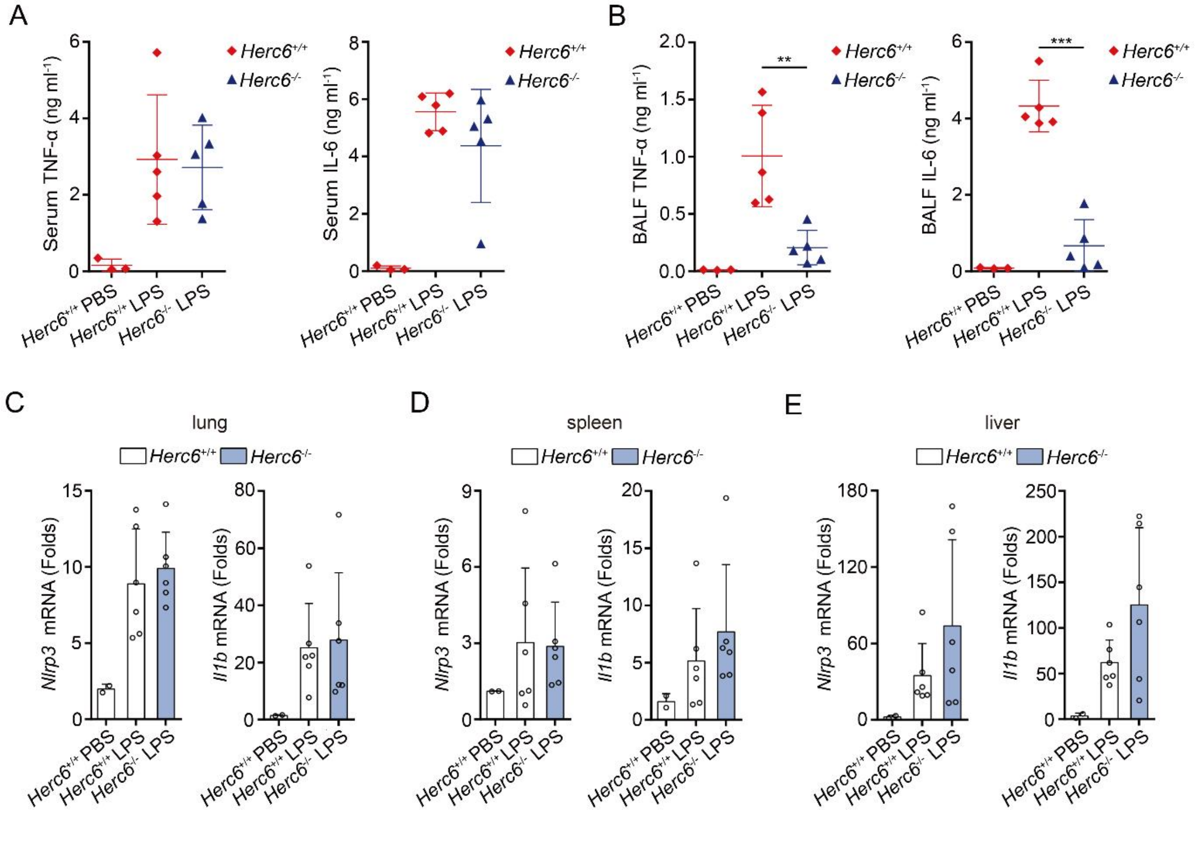

Supplementary Figure 6. *Herc6* deficiency ameliorates inflammation in vivo

(**A** and **B**) ELISA analysis of serum (**A**) or BALF (**B**) levels of TNF-α and IL-6 of *Herc6*$^{+/+}$ or *Herc6*$^{-/-}$ mice after i.p. LPS injection for 12h (PBS, n=3; LPS, n=5 per group, two-tailed *t*-test *Herc6*$^{+/+}$ vs. *Herc6*$^{-/-}$, **B**: **p = 0.005067, ***p =2.89×10^{-5}).

(**C**, **D,** and **E**) RT-PCR analysis of *Nlrp3* and *Il1b* in the lung (**C**), spleen (**D**), and liver (**E**) of *Herc6*$^{+/+}$ or *Herc6*$^{-/-}$ mice after i.p. LPS injection for 12h (PBS, n=2; LPS, n=6 per group).

All data are represented as mean ± SD. Similar results were obtained from three independent experiments.

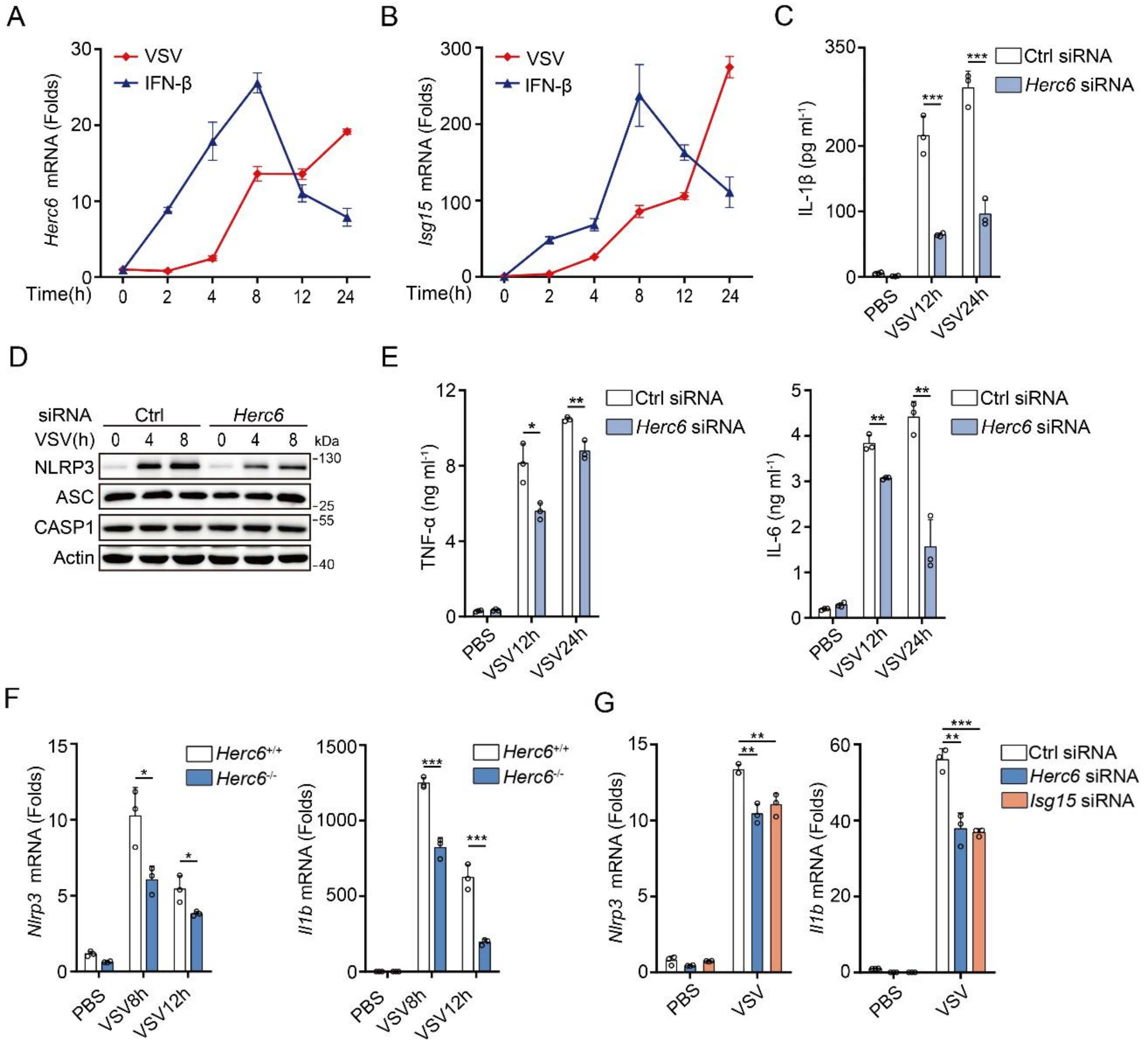

Supplementary Figure 7. HERC6 promotes VSV-induced inflammation

(**A** and **B**) RT-PCR analysis of *Herc6* (**A**) or *Isg15* (**B**) mRNA in VSV-infected or mIFN-β-stimulated mouse PMs.

(**C**) ELISA analysis of IL-1β in supernatants from mouse PMs transfected with Ctrl or *Herc6* siRNA for 48h, following VSV infection for 12 or 24 h (two-tailed *t*-test Ctrl vs. *Herc6* siRNA, ***p = 0.000909, ***p = 0.000556).

(**D**) Immunoblot analysis of mouse PMs transfected with Ctrl or *Herc6* siRNA for 48h following VSV infection.

(**E**) ELISA analysis of TNF-α and IL-6 in supernatants from mouse PMs transfected with Ctrl or *Herc6* siRNA for 48h, following VSV infection for 12 or 24 h (two-tailed *t*-test Ctrl vs.

Herc6 siRNA, TNF-α: *p = 0.017593, **p = 0.005113; IL-6: **p = 0.001831, 0.002013).

(**F**) RT-PCR analysis of *Nlrp3* and *Il1b* mRNA in VSV-infected mouse PMs from *Herc6*$^{+/+}$ or *Herc6*$^{-/-}$ mice for 8h or 12h (two-tailed *t*-test *Herc6*$^{+/+}$ vs. *Herc6*$^{-/-}$, *Nlrp3*: *p = 0.024776, 0.034645; *Il1b*: ***p = 0.000612, 0.000984).

(**G**) RT-PCR analysis of *Nlrp3* and *Il1b* mRNA in mouse PMs transfected with Ctrl, *Herc6,* or *Isg15* siRNA for 48h, following VSV infection for 8h (two-tailed *t*-test Ctrl vs. *Herc6* siRNA, *Nlrp3*, **p = 0.002537; *Il1b*, **p = 0.003381; two-tailed *t*-test Ctrl vs. *Isg15* siRNA, *Nlrp3*, **p = 0.007935; *Il1b*, ***p = 0.000414).

All data are represented as mean ± SD in A, B, C, E, F, and G. Similar results were obtained from three independent experiments.

Supplementary Table 1 The possible ubiquitination sites of NLRP3.

Residus	Score	Ubiquitinated	Residus	Score	Ubiquitinated
2	0.08	No	430	0.77	Yes, Medium confidence
9	0.34	No	437	0.6	No
23	0.35	No	481	0.37	No
24	0.4	No	496	0.59	No
26	0.32	No	510	0.59	No
36	0.57	No	518	0.51	No
48	0.53	No	540	0.54	No
66	0.47	No	552	0.36	No
86	0.62	Yes, Low confidence	567	0.47	No
88	0.59	No	570	0.37	No
93	0.8	Yes, Medium confidence	594	0.32	No
131	0.18	No	595	0.28	No
133	0.16	No	599	0.32	No
134	0.15	No	610	0.31	No
138	0.14	No	615	0.29	No
139	0.1	No	617	0.26	No
142	0.13	No	619	0.26	No
166	0.24	No	620	0.24	No
175	0.28	No	652	0.49	No
192	0.75	Yes, Medium confidence	689	0.88	Yes, High confidence
194	0.7	Yes, Medium confidence	698	0.8	Yes, Medium confidence
204	0.58	No	799	0.68	Yes, Low confidence
232	0.33	No	823	0.45	No
238	0.28	No	830	0.31	No
289	0.23	No	831	0.34	No
293	0.18	No	878	0.48	No
324	0.71	Yes, Medium confidence	880	0.5	No
338	0.39	No	888	0.26	No
339	0.42	No	927	0.47	No
357	0.32	No	930	0.39	No
377	0.42	No	942	0.56	No
379	0.32	No	973	0.59	No
384	0.55	No	994	0.42	No
423	0.44	No	1015	0.68	Yes, Low confidence
			1025	0.64	Yes, Low confidence

Supplementary Table 2 Sequences of PCR primers used in this study

Name	Prime	Sequence
m*Actb*	Forward	5’-TGTTACCAACTGGGACGACA-3’
	Reverse	5’-CTGGGTCATCTTTTCACGGT- 3’
m*Nlrp3*	Forward	5'-TGGATGGGTTTGCTGGGAT-3'
	Reverse	5'-CTGCGTGTAGCGACTGTTGAG-3'
m*Il1b*	Forward	5'-ACCTTCCAGGATGAGGACATGA-3'
	Reverse	5'-AACGTCACACACCAGCAGGTTA-3'
m*Tnfa*	Forward	5'-GCCACCACGCTCTTCTGTCT-3'
	Reverse	5'-TGAGGGTCTGGGCCATAGAAC-3'
m*Il6*	Forward	5'-ACAACCACGGCCTTCCCTAC-3'
	Reverse	5'-CATTTCCACGATTTCCCAGA-3'
m*Isg15*	Forward	5′-AGAAGCAGATTGCCCAGAAG-3′
	Reverse	5′-TGCGTCAGAAAGACCTCATAGA-3′
m*Herc6*	Forward	5′-ACCAGCGCATTGAACTCTT-3′
	Reverse	5′-GAATTGGTTCTGGCCTTTCGG-3′
h*NLRP3*	Forward	5′-TCCAGCATCCTGGCTGTAAC-3′
	Reverse	5′-GGGAATGGCTGGTGCTCAATA-3′
h*HERC5*	Forward	5′- GACGAACTCTTGCACCGTCT -3′
	Reverse	5′- CCTCAATTGCTGCCGACCTA -3′
h*ACTIN*	Forward	5′- GGAAATCGTGCGTGACATTAA -3′
	Reverse	5′- AGGAAGGAAGGCTGGAAGAG -3′
